# Nanomedicine and drug delivery to the retina: current status and implications for gene therapy

**DOI:** 10.1007/s00210-022-02287-3

**Published:** 2022-09-15

**Authors:** Mohamed Tawfik, Fang Chen, Jeffrey L. Goldberg, Bernhard A. Sabel

**Affiliations:** 1 Institute of Medical Psychology, Medical Faculty, Otto-Von-Guericke University, Magdeburg, Germany; 2 Spencer Center for Vision Research, Byers Eye Institute, Stanford University School of Medicine, Palo Alto, CA USA

**Keywords:** Retinal drug delivery, Nanomedicine, Retina, Retinal diseases, Gene therapy, Nanoparticles

## Abstract

Blindness affects more than 60 million people worldwide. Retinal disorders, including age-related macular degeneration (AMD), diabetic retinopathy (DR), and glaucoma, are the leading causes of blindness. Finding means to optimize local and sustained delivery of drugs or genes to the eye and retina is one goal to advance the development of new therapeutics. Despite the ease of accessibility of delivering drugs via the ocular surface, the delivery of drugs to the retina is still challenging due to anatomic and physiologic barriers. Designing a suitable delivery platform to overcome these barriers should enhance drug bioavailability and provide a safe, controlled, and sustained release. Current inventions for posterior segment treatments include intravitreal implants and subretinal viral gene delivery that satisfy these criteria. Several other novel drug delivery technologies, including nanoparticles, micelles, dendrimers, microneedles, liposomes, and nanowires, are now being widely studied for posterior segment drug delivery, and extensive research on gene delivery using siRNA, mRNA, or aptamers is also on the rise. This review discusses the current state of retinal drug/gene delivery and highlights future therapeutic opportunities.

## Introduction

Vision is considered the key enabling sense for a person to work and function independently. Therefore, the eye is the most important sensory organ and the one that people most fear losing (Chiang et al. 2006). Because losing vision will severely impair quality of life, it is a global burden, especially given the rising prevalence of blinding ocular diseases in our aging populations. The number of visually impaired people worldwide is estimated to be 253 million with an estimated 25% increase in the incidence of blindness and visual impairment by 2030 (Ackl et al. 2017).

To date, there are novel options to protect or restore vision, but little is available to inhibit retinal neurodegeneration (Sabel et al. 2011; Dundon et al. 2015; Gall et al. 2016). One of the reasons for the lack of progress in this field is the difficulty of delivery of potential therapeutic agents to the posterior segment of the eye, including anatomic/physical barriers such as the blood-aqueous barrier and the blood-retinal barrier (Awwad et al. 2017). Overcoming these barriers using drug delivery technology for the targeting of neuroprotective agents to the posterior eye promises to not only improve patients' lives and reduce healthcare costs but may also provide a better understanding of the treatment and management of other neurodegenerative diseases (Zhang et al. 2012; Albrecht et al. 2020).

**Fig. 1:**
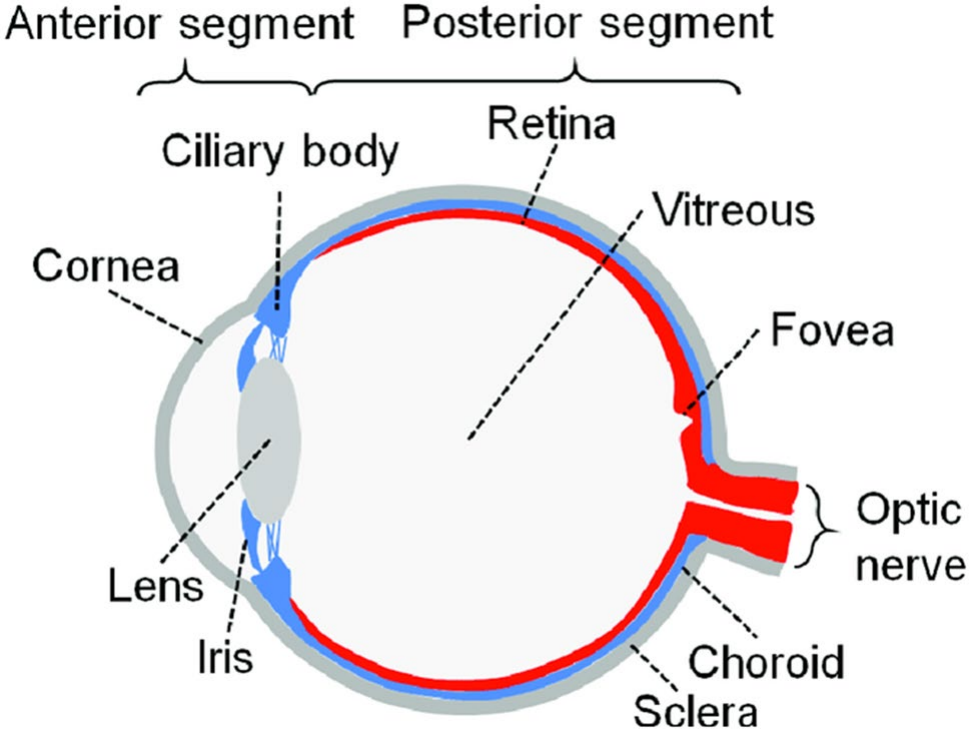
Diagram of the human eye. The anterior segment of the eye contains the cornea, iris, ciliary body, and lens. The posterior segment of the eye consists of the vitreous humor, retina, choroid, and optic nerve. Reproduced with permission (Chuang et al. 2017)

The eye is divided into two compartments: the anterior and the posterior segments. The anterior segment includes the cornea, iris, pupil, ciliary body, and conjunctiva; the posterior segment includes the sclera, choroid, fovea, vitreous humor, optic nerve, and retina (Bajpai et al. 2016) (Fig. 1).

Visual information from our environment is focused on the retina, where light signals are translated to neuronal signals for preprocessing by the retina and sending neuronal signals down the optic nerve to the brain. The retina, therefore, plays a crucial role in obtaining and analyzing finely detailed visual acuity, color, and motion detection, among other visual features which are then further processed by the brain (Masl 2001; Arslan 2018). Due to the poor regenerative capacity of the neural retina after damage, this often causes long-lasting vision impairment and blindness of diseases such as glaucoma, age-related macular degeneration (AMD), and diabetic retinopathy (DR) (Arslan 2016; Gaudana et al. 2009). To reduce the damage, halt progression, or enhance regenerative/restorative processes, different obstacles and biological barriers have to be overcome to deliver drugs and other therapeutic agents. This research field, generally referred to as “drug delivery,” has witnessed tremendous growth in recent years with many innovative techniques to target retinal tissue. In this review, we highlight such innovations in retinal drug delivery, leveraging nanomedicine and other techniques such as viral vectors and non-viral gene therapy.

## Barriers to retina drug delivery

**Fig. 2:**
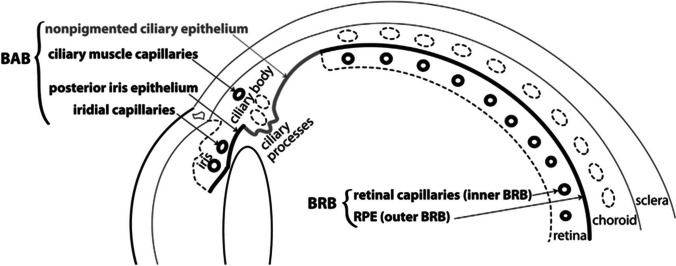
Blood-ocular barriers. The thicker lines and black text indicate tight endothelium/epithelium; dashed lines and gray text indicate leaky endothelium/epithelium. Reproduced with permission (Del Amo et al. 2017)

The best established and most widely used ocular treatments are topical in the form of eye drops. However, topical drug absorption is limited by a variety of ocular barriers which regulate and limit the absorption rate in the anterior and posterior segments of the eye, hence reducing drug bioavailability. These barriers include the tear film, cornea, conjunctiva, sclera, blood-aqueous barrier, and blood-retina barrier (Del Amo et al. 2017) (Fig. 2).

### Tear film

The tear film is the fluid wrapping the surface of the cornea between the lid margins with a thickness of only 3–10 μm. While it is easy to apply, the secretion and turnover of tears dilute the drug concentration, and the drug is drained through the nasolacrimal duct, diminishing its ocular residence time (Korb et al. 2002; Winter et al. 2010).

### Cornea

The cornea limits the transport of ocular drugs into the eye, and it is composed of five layers: the corneal epithelium, basement membrane, stroma, Descemet’s membrane, and corneal endothelium. The cornea hardly allows any transportation of drugs across its cellular layers, and drug transport is affected by different factors, such as lipophilicity/hydrophilicity, molecular weight, surface charge, and the degree of ionization of the drug. Additionally, the aqueous humor, the anterior lens, and the vitreous humor limit drug delivery to the posterior segment (Dua et al. 2013; Malhotra et al. 2001).

### Conjunctiva

This is a layer of mucous membrane that consists of an outer epithelial and goblet cell layer. Due to the highly vascularized structure of the conjunctiva, the majority of the drugs delivered to the eye directly enter the systemic circulations (Barar et al. 2009).

### Sclera

The sclera is made of collagen and polysaccharides. Because it is less vascularized than the conjunctiva, it permits higher drug permeation than the cornea and almost half of the conjunctival permeation (Hämäläinenet al. 1997).

### Blood-aqueous barrier

The blood-aqueous barrier (BAB), located in the anterior eye segment, is made up of the epithelium tissue of non-pigmented ciliary and endothelial cells of the iris vascular system. Both have tight intersections which limit the delivery of drugs from the systemic circulation toward the anterior segment. The BAB hinders the permeability of macromolecules such as plasma albumin across the aqueous humor. Smaller lipophilic molecules can more easily infiltrate and they are generally eradicated through the uveal tract (Freddo, 2001).

### Blood-retinal barrier

The blood-retinal barrier (BRB) is composed of an inner barrier and an outer barrier. Retinal capillary endothelial cells and their connections form the inner blood-retinal barrier. The retinal pigment epithelium (RPE) and the Bruch’s membrane form the outer blood-retinal barrier which separates the retinal tissue from choroidal circulation. This is processed by specialized transport mechanisms such as passive diffusion, active transport, and efflux pumps, limiting the transport of drugs across the systemic circulation toward the posterior segment (Cunha-Vaz 1976).

## Routes of drug delivery to the retina

**Fig. 3:**
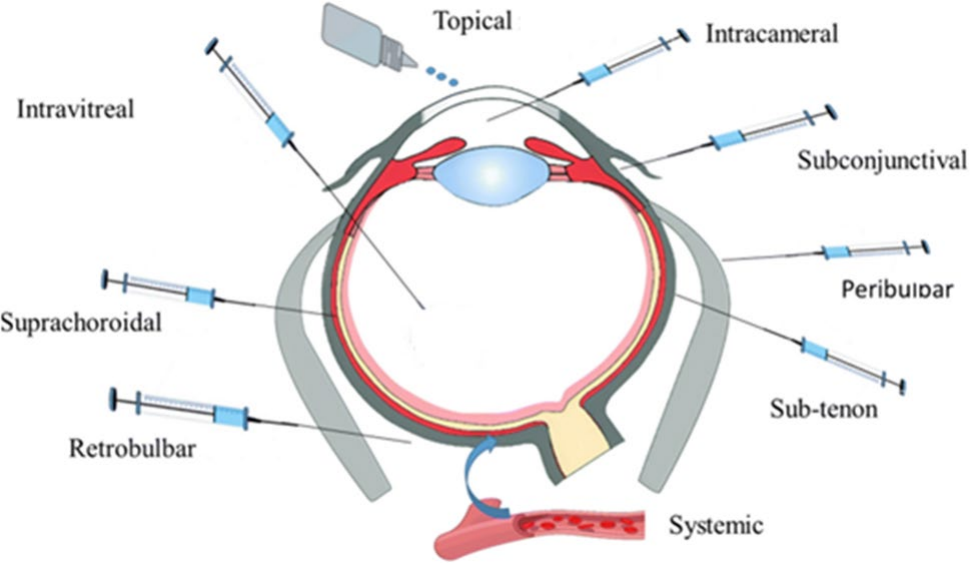
Routes of drug administration for ocular delivery. Adapted from reference (Gaballa et al. 2021)

There are two fundamental routes of drug delivery to the retina: the invasive and non-invasive route (Fig. 3). Table 1 summarizes the advantages and disadvantages of the various routes of drug delivery to the retina.

**Table 1:** Summary table of advantages and disadvantages of the various routes of drug delivery to the retina

Delivery routes	Advantages	Disadvantages
A. Non-invasive routes:		
Topical route	Easy to administer	Poor retention; poor efficiency; requires frequent administration
Iontophoresis	High tissue penetration of the drug	Complexed drug release system; risk of skin damage
B. Invasive local routes:		
Intravitreal injection	Direct injection into the vitreous; easy to reach the retina	Highly invasive; risk of endophthalmitis, hemorrhage, cataracts, and retinal detachment; requires a proficient practitioner
Subretinal injection	Direct contact with the photoreceptor and RPE	Risk of retinal detachment; retinotomy; involves vitrectomy; limited treatment location; complexity in administration
Suprachoroidal (SC) injection	Less invasive; great surface-area coverage; requires no anesthesia	Rapid clearance by the choriocapillaris; compromised efficiency
C. Periocular routes:		
Subconjunctival injection	Minimally invasive; no surgical facility needed; no post-injection interventions needed	Temporary subconjunctival hemorrhage and local pain
Peribulbar (PB) injection	Safe; easy to apply; ideal for anesthesia	Requires multiple injections; a large volume is needed
Retrobulbar (RB) injection	Ideal for anesthesia	Risk of optic nerve damage
Sub-tenon (ST) injection	No sharp needles; less risky; effective anesthesia	Minor complications associated
D. Systemic routes:		
Oral route	Non-invasive, easy to administer	Poor efficiency; hard to reach the retina; need a high dose
Intravenous route	Non-invasive to the eye	Patient selective; low bioavailability

### Non-invasive

#### Topical route

The topical route is the most common and patient-friendly path and is used for the treatment of both segments. However, topical formulations fail to deliver enough drugs to the retinal tissues for many different reasons: limited contact time with the ocular surface, the long diffusion path from the cornea to retinal tissue, dilution by tear turnover, nasolacrimal drainage, protein binding, and systemic absorption in the precorneal area. Therefore, topical formulations of even highly effective drugs are rather inefficient for retinal diseases. Ways to compensate for this poor delivery are frequent drug administration (up to 8 times daily), yet only 50% of glaucoma patients use their eye drops properly. Despite the plausible ease of administration, patients with serious vision loss have difficulty reliably using eye drops (Urtti et al. 1990; Subrizi et al. 2019).

#### Iontophoresis

Ocular iontophoresis uses low current flow to deliver drugs through the ocular barriers. This can be achieved through electroporation, electrophoresis, or electro-osmosis. For posterior segment treatment, the trans-scleral pathway is used to avoid anterior segment barriers. Aciont Inc. has initiated a phase 1 clinical trial for the treatment of AMD by a Visulex-1-noninvasive ocular drug device to deliver the drugs bevacizumab and ranibizumab. Although this route can avoid the associated effects of invasive procedures, the current intensity may carry a risk of damage due to the prolonged exposure of the skin to an electrical current (Molokhia et al. 2007; Chopra et al. 2012; Souza et al. 2014).

### Invasive delivery

#### Intravitreal injection

Intravitreal injections are the preferred route for posterior segment treatment as it overcomes most limiting mechanisms and barriers. Here, the therapeutics are injected directly into the vitreous to achieve the required therapeutic concentration. Intravitreal drugs easily reach the vitreous cavity and retina: they can spread through the entire vitreous humor and reach the retina within several hours after injection. However, the drug diffusion from the vitreous into the retina is limited by the internal limiting membrane (ILM), and the drug permeation from the vitreous cavity to the choroid is slow due to RPE obstruction. The drug concentration in the vitreous cavity is influenced by the initial dosage, delivery volume, and removal rate. Furthermore, the diffusivity of negatively charged molecules in the vitreous humor was found to be superior to that of positively charged ones. However, frequent injections are usually required for this route with risks for adverse events including pain, infection, endophthalmitis, intraocular inflammation, elevated intraocular pressure (IOP), retinal detachment, and cataract formation which are not always communicated properly with the patient (Rowe-Rendleman et al. 2014; Edelhauser et al. 2010; Maurice 2001).

#### Subretinal injection

The subretinal area is located between the RPE and the photoreceptors. Hence, administrated drugs via this route will be in direct contact with the photoreceptor’s membrane, RPE cells, rendering it to be the preferred site for drug delivery for the treatment of retinal diseases. This route of delivery is surgically tricky and at this point mainly used for gene therapy of retinal degenerative diseases. Because this option is invasive, it may cause temporary focal retinal detachment and the creation of a retinotomy, posing higher risks for patients whose retinal cellular integrity has already been compromised. Moreover, the vitrectomy procedure, which facilitates subretinal administration, carries a high risk of inducing cataracts and a low risk of retinal detachment. Another limitation of the subretinal delivery approach is that the spread of the delivered vectors to the subretinal space is minimal, mostly limited to the area at or near the injection site. Therefore, subretinal delivery may result in suboptimal therapeutic benefits for diseases that benefit most from diffuse transduction of peripheral retinal signals, such as retinitis pigmentosa (Peng et al. 2017; Simunovic et al. 2017; Ghazi et al. 2016).

#### Suprachoroidal injection

The suprachoroidal (SC) route is a relatively new mode of drug administration that was introduced in 2002 and firstly used in humans in 2013. Such injections are made under the conjunctival membrane that lines the inner surface of the eyelid, avoiding the cornea and conjunctiva, targeting the suprachoroidal space mainly with microneedles that are typically 1000 μm in length with 50 µL injection volumes. In this way drugs can travel through the sclera and into the posterior section, avoiding direct entry to the inner eye and hence decreasing the chance for endophthalmitis and retinal detachment. Unlike subretinal delivery, suprachoroidal delivery does not require retrobulbar anesthesia in an operating room. Moreover, suprachoroidal delivery offers the potential for greater surface-area coverage of the posterior segment compared to focal subretinal administration. Unlike intravitreal administration, suprachoroidal delivery is not hindered by the internal limiting membrane or the potential for particles/floaters in the visual axis. Suprachoroidal drug delivery, however, may face several challenges. Effective transduction of the retina after suprachoroidal administration could be hindered by rapid clearance by the choriocapillaris, although the choroidal vascular pore size may limit the entry of vectors or nonviral nanoparticles. Finally, intravitreal injection of bevacizumab showed a more sustained pharmacologic profile than does a similar dose delivered to the suprachoroidal space. Intravitreal injections are distributed more in the inner retina, whereas suprachoroidal delivery occurs primarily at the choroid, retinal pigment epithelium, and photoreceptor outer segments (Olsen et al. 2011; Patel et al. 2012, 2011; Rai et al. 2015; Olsen et al. 2011).

#### Periocular route

The periocular route refers to the use of space surrounding the eyeball but within the orbit to deliver drugs. It is a potentially safe alternative for intravitreal injection with four sub-routes: subconjunctival, peribulbar, retrobulbar, and sub-tenon.

#### Subconjunctival injection

The subconjunctival space is just beneath the conjunctiva membrane which covers the sclera and is easier to reach in comparison with topical administration. It has long been used clinically to deliver drugs to the anterior segment. Experimentally, larger particles (200 nm) are retained for longer periods in subconjunctival space in comparison to smaller particles (20 nm). Suprachoroidal injections may result in the highest bioavailability as demonstrated by a higher delivery rate of sodium fluorescein to choroid-retina when compared to intravitreal and posterior subconjunctival injections (Raghava et al. 2004; Waite et al. 2017; Tyagi et al. 2012).

#### Peribulbar injection

The peribulbar (PB) pathway entails injections above and/or below the globe. This path is ideal for delivering anesthesia during cataract surgery. In comparison to anesthetic deposited outside the muscle cone, PB has a lower chance of damaging intra-orbital structures (Johnson and Chu 2010; Iwao et al. 2007).

#### Retrobulbar injection

The retrobulbar (RB) route entails drug injecting directly into the retrobulbar room via the eyelid and orbital fascia. However, it is an operation that runs the risk of causing damage to the optic nerve. This path, on the other hand, is mainly used to transport anesthetics that can induce intraocular pressure changes (Okada et al. 2003; Pautler et al. 1986; Davis and Mandel 1986).

#### Sub-tenon (ST) injection

Sub-tension (ST) injection places drugs into the cavity between the tenon's capsule and the sclera. For dosage management, this method necessitates the use of a blunt cannula. The sub-tenon injection can bypass the conjunctival and orbital vessels and lymphatic supply. Hence, it is thought to be a safer route for providing anesthesia than the retrobulbar and peribulbar routes due to fewer risks and the lack of sharp needles (Ogura et al. 2019; Maeda et al. 2019; Moshfeghi et al. 2002; Lee et al. 2008). Using sub-tenon injection, Kalita et al. showed increased facilitated trans-scleral transport of polymethylacrylate nanoparticle loaded with carboplatin (anti-cancer drug), with a sustained-release release profile without any associated short-term ocular or systemic side effects in six patients. The highest level of carboplatin was detected in retinas up to 24 h post-treatment. The intravitreal concentration continued to increase gradually until 72 h. The choroids and lenses showed very low levels of carboplatin after 6 h, with negligible amounts at 72 h. Despite the positive results, further studies are needed to assess long-term toxicity and clinical efficacy (Kalita et al. 2014).

#### Systemic administration

Systemic administration is achieved by drug delivery using the oral or intravenous route. The systemic route is not effective in delivering drugs to retinal tissues because the BRB restricts the ocular entry of therapeutic molecules from the systemic circulation, by keeping the drugs isolated in the choroid without reaching the retina. The vitreous humor volume is about 4–5 mL, while the entire blood volume is in the order of 5 L. This creates a significant dilution effect for drugs being delivered via the systemic route. Consequently, drugs administered systemically have very low bioavailability in the order of only 2%. Therefore, relatively high systemic doses are required to achieve adequate drug concentrations in the eye, which can lead to systemic side effects and adverse events. On the other hand, there are studies reporting the efficacy of bevacizumab (anti-VEGF for the treatment of AMD) administered via the intravenous route. One clinical study has shown that the systemic route of bevacizumab may improve visual acuity without significant systemic side effects in AMD and DR patients with no history of hypertension or thrombosis. With the increase in colloidal delivery system innovations such as nanoparticles, this route could provide an alternative route of administration for patients who are non-compliant with invasive administration (Raviola 1977; Occhiutto et al. 2012; Meyer and Holz 2011).

In sum, there are many options for delivering drugs to the eye for different retinal disease targets, and their usability depends on their respective bioavailability and associated risk profiles.

## Retinal diseases

### Diabetic retinopathy

Diabetic retinopathy (DR) is the leading cause of blindness and visual impairment in working-age individuals. In 2012, 93 million people were estimated to have DR (Yau et al. 2012). It is defined by a lack of adequate oxygen and nutrient supply to the retina to sustain proper eye health and function. Among the underlying pathophysiology, chronic retinal ischemia leads to vision impairment, as well as compensatory neovascularization, again linked to vascular endothelial growth factor upregulation. These new blood vessels are permeable and may also break more easily, resulting in vascular leakage and retinal hemorrhage, disrupting vision even further. Hemorrhage causes scarring and eventually retinal detachment, resulting in profound vision loss if left untreated. Prevention of DR is most critical and relies on blood sugar and blood pressure control. Once DR is present, treatment options are limited. Laser photocoagulation to ablate the peripheral retina reduces ischemic drive for neovascularization; as in AMD, anti-VEGF therapy prevents vascular leakage, and chronic steroid supplementation can also help (Patel et al. 2008; Adamis et al. 1994; Wilkinson et al. 2003).

### Age-related macular degeneration

Age-related macular degeneration (AMD) is a leading cause of central vision loss, particularly in people over the age of 65, with estimates of prevalence as high as 288 million patients by 2040. AMD is characterized by an abnormality in and degeneration of the RPE, which leads to photoreceptor cell death in the macula and consequent vision loss. To date, AMD pathophysiology is poorly understood. However, the pathophysiology includes an accumulation of drusen underneath the RPE, yellowish extracellular deposits of lipids and protein which may result from and/or cause RPE dysfunction, and changes in Bruch membrane permeability. Early AMD is characterized by drusen without significant loss of vision (dry AMD). Advanced AMD leads to loss of vision and can be divided into two categories: degeneration of the RPE, so-called geographic atrophy, and choroidal neovascularization (CNV), so-called wet AMD. Anti-VEGF agents delivered by intravitreal injection are the gold standard therapeutics for wet AMD. Oral supplements including vitamins have been demonstrated to slow the course of moderate to severe dry AMD. There are genetic loci that confer significant risk in the development of AMD including complement factor H (CFH), which are now under investigation as therapeutic targets (Table 2) (Wong et al. 2014a, b; Green and Key 1977; Johnson et al. 2001; Gehrs et al. 2006; Hollyfield et al. 2008; Ferrara 2010; Ambati and Fowler 2012; Ding et al. 2009).

**Table 2:** Summary table on established and experimental therapies for retinal diseases

Retinal diseases	Established and experimental therapies*
Diabetic retinopathy (DR)	• Blood sugar and blood pressure control to prevent DR • Laser photocoagulation to reduce neovascularization • Injection of anti-VEGF to prevent vascular leakage • Eye surgery to remove partial vitreous humor • Chronic steroid supplementation
Age-related macular degeneration (AMD)	• Intravitreal injection of anti-VEGF medicines (ranibizumab, aflibercept, and brolucizumab) to treat wet AMD • Oral supplements of vitamins • Photodynamic therapy (PDT) destroys the abnormal blood vessels that cause wet AMD
Glaucoma	• Prescription eyedrops (prostaglandins, beta blockers, alpha-adrenergic agonists, carbonic anhydrase inhibitors, rho kinase inhibitors, miotic or cholinergic agents) to decrease eye pressure • Oral medications, usually a carbonic anhydrase inhibitor • Laser trabeculoplasty to treat open-angle glaucoma • Trabeculectomy to remove part of the trabecular meshwork • Drainage tubes to lower eye pressure • Minimally invasive glaucoma surgery to lower eye pressure • Laser peripheral iridotomy to treat acute angle-closure glaucoma • Cell transplantation to repair or replace damaged retinal tissues. *

### Glaucoma

Glaucoma is defined by progressive optic nerve damage with characteristic structural and functional features. Glaucoma is the most common cause of irreversible visual field loss in the world. It is associated with a number of etiologies and pathophysiology, including increased intraocular pressure (IOP), optic nerve ischemia, and vascular dysregulation which cause activation of oxidative stress-related responses. Drugs (mostly applied as topical eye drops) and surgical procedures, such as laser and microsurgery, aim mostly at lowering IOP in treating glaucoma. Despite early identification and quick access to pharmacotherapy, visual field decline may continue. Neurodegeneration and visual field loss are also targets of new neurotherapeutic approaches which aim at improving visual field functions through non-drug therapies such as vision training, non-invasive brain stimulation, and delivery of neurotrophic factors (Tham et al. 2014; Zhang et al. 2021; Almasieh et al. 2012; Weinreb et al. 2014; Sabel and Gudlin 2014; Sabel et al. 2018; Sabel et al. 2020a, b). Preclinically, cell therapies including RGC transplantation and stem cell therapies have been used to replace damaged retinal tissues (Chao et al. 2017; Lee et al. 2021; Venugopalan et al. 2016; Shirai et al. 2016; Gonzalez-Cordero et al. 2013; Hambright et al. 2012; Zhang et al. 2020a, b).

## Novel strategies and approaches to enhance retina drug delivery

**Fig. 4:**
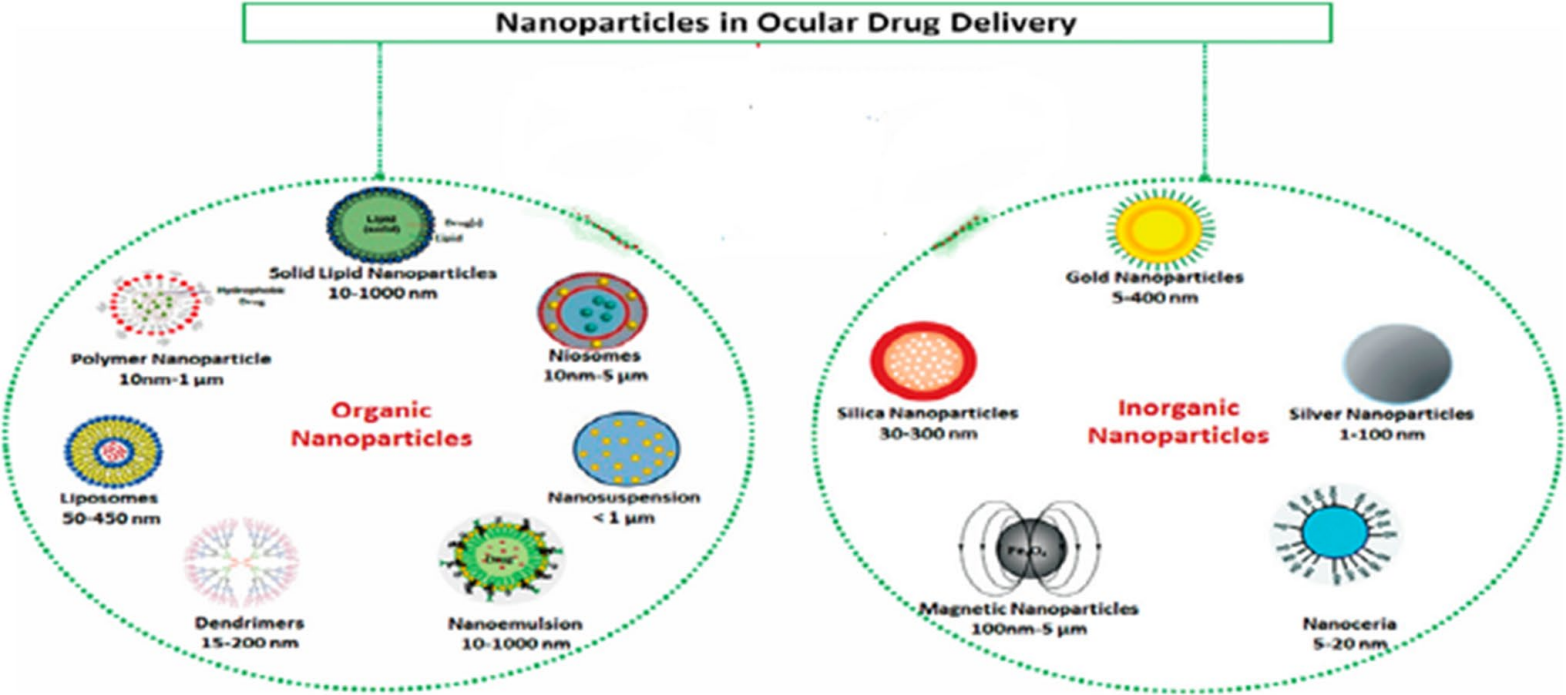
Schematic drawings for potential organic and inorganic nanoparticles for ophthalmic drug delivery. Adapted from reference (Meza-Rios et al. 2020)

In recent years different ophthalmic formulations were tested for retinal drug delivery so that drugs or genes can bypass ocular barriers. The progress of novel drug delivery approaches is summarized in Table 3. They include nanoparticles, liposomes, dendrimers, micelles, microneedles, ocular implants/inserts, and contact lenses (Desai et al. 2019). The chemical structures of representative chemicals used for improving drug delivery are shown in Fig. 4.

**Table 3:** Summary table on the novel drug delivery approaches for retinal diseases

Drug delivery systems (DDS)	Applications in retinal diseases treatment and concerns
A. Nanomedicine	
Liposomes	• Visudyne to treat AMD • Tears again™ to treat dry eye • Deliver Tacromilus to treat autoimmune uveoretinitis • Deliver dexamethasone to the posterior eye chamber after topical administration • Deliver paclitaxel to counteract choroidal neovascularization after intravenous injections • Deliver latanoprost to reduce IOP after a single subconjunctival injection • Potential risks: blurred vision, inflammatory response, low physical stability, and poor controlled and sustained release
Dendrimers	• Efficiently deliver brimonidine tartrate to treat glaucoma through topical administration • Deliver carboplatin to suppress tumor vasculature after subconjunctival injection • Deliver acetazolamide to reduce the IOP • Deliver fluocinolone acetonide (TA) to treat neuroinflammation in retinal degeneration • Halt retinal degeneration while preserving the photoreceptors after intravitreal injection • Drawbacks: high toxicity profile
Micelles	• Encapsulate and enhance pharmacokinetics of hydrophobic drugs for the treatment of posterior eye diseases • Deliver poly-L-lysine to treat CNV by intravenous injection • Deliver pazopanib with 25% efficiency and no toxicity in retinal cells after intravitreal injection
Polymer nanoparticles	• PEG: Pegaptanib (pegylated aptamer) functions as anti-angiogenic medicine for the treatment of neovascular AMD (clinical approval) • PVP: artificial vitreous substance; scaffolds for lens regeneration; deliver antiglaucoma drugs; cross the BRB to deliver small hydrophobic molecules after intravenous injection • Potential risks of PVP: hazy cornea, intravitreal opacity, and inflammation • PLA, PGA, PLGA: deliver betamethasone phosphate through intravenous injection; deliver plasmid to the retina after intravitreal injection; sustained-release brinzolamide through subconjunctival injection; sustained-release dexamethasone after intravitreal injection • PCL: sustained release of dexamethasone for one year; increase the bioavailability of cyclosporine • PBCA: across the BBB and BRB for drug delivery • Overall concerns: need to scale up, insufficient toxicological assessments
Inorganic nanomaterials	• Magnetic nanoparticles: deliver BDNF/NGF into the retina for neuroprotection through intravitreal injection; target RPE with magnetic nanoparticles • Cerium oxide nanoparticles could down-regulate caspase-induced apoptosis in the retina and protect retinal cells from ROS-induced damage after intravitreal injection • Silica-nickel/iron hybrid micro-propeller to rapidly deliver concentrated cargo to a defined region at the posterior pole of the eye • Concern: need more investigations into biodegradability, clearance, and toxicity
B. Ocular inserts	
ForSight VISION5	• Topical delivery of prostaglandin like-drugs for glaucoma, dry eye, and allergy treatment
AP-PCL inserts	• Sustained delivery of brimonidine tartrate through subconjunctival route
Ocular implants	• Ozurdex (Allergan): dexamethasone implant for macular edema treatment • Durasert (pSivida Corp): sustained drug release from days to years • Vitrasert: intravitreal delivery of antiviral drug-ganciclovir for the treatment of cytomegalovirus retinitis • Retisert (Bausch + Lomb): releases TA up to three years into the vitreous humor to treat posterior uveitis • Iluvien: (TA intravitreal implant) intravitreal injectable insert for the treatment of DME • I-vation TA (SurModics Inc.): intravitreal drug delivery implant for triamcinolone acetonide (TA) sustained release
Hydrogels	• Subconjunctival injected ocular inserts, topical eyedrops, and combination systems with nanocarriers • Intravitreal injection of hydrogel to retain bevacizumab in rabbit eyes • Composite DDS: chitosan-based gel containing colloidal nanocarriers sustain IOP reduction for 40 days in a rabbit glaucoma model; injectable PNIPAAm-based thermo-responsive hydrogel with PLGA microspheres to encapsulate ranibizumab or aflibercept and release them in a controlled manner for ~ 200 days; collagen shield containing titania and zinc oxide to sustained release pilocarpine hydrochloride
Contact lenses	Increase residence time of drugs on the cornea • Timolol-loaded nanoparticles within a silicone hydrogel contact lens reduced IOP for 192 h in a rabbit model • Molecularly imprinted lenses reduced IOP • Surfactant-coated contact lenses increased the release duration of dexamethasone 21-disodium phosphate from about 2 to 50 h • Contact lenses have high surface charge increase the loading and extend the release duration of ionic compounds • Collagen shields to sustained release drugs, increase drug concentrations, and lubricate the eye when they dissolve • Drawbacks: inadequate handling and low patient compliance, risks in dry eye and ocular surface infections
Microneedles	• Suprachoroidal CLS-TA injector (Clearside Biomedical Inc.) to treat macular edema associated with non-infectious uveitis • Concern: potential risk in increasing IOP
Microelectromechanical system	• Electrolysis-based controlled drug delivery • Drawback: potential side effects due to a minimally invasive procedure
Encapsulated cell technology	• Renexus: ocular implantation of human RPE transfected with a plasmid encoding CNTF to treat dry AMD, glaucoma, and retinitis pigmentosa

### Nanomedicine in retina drug delivery

**Fig. 5:**
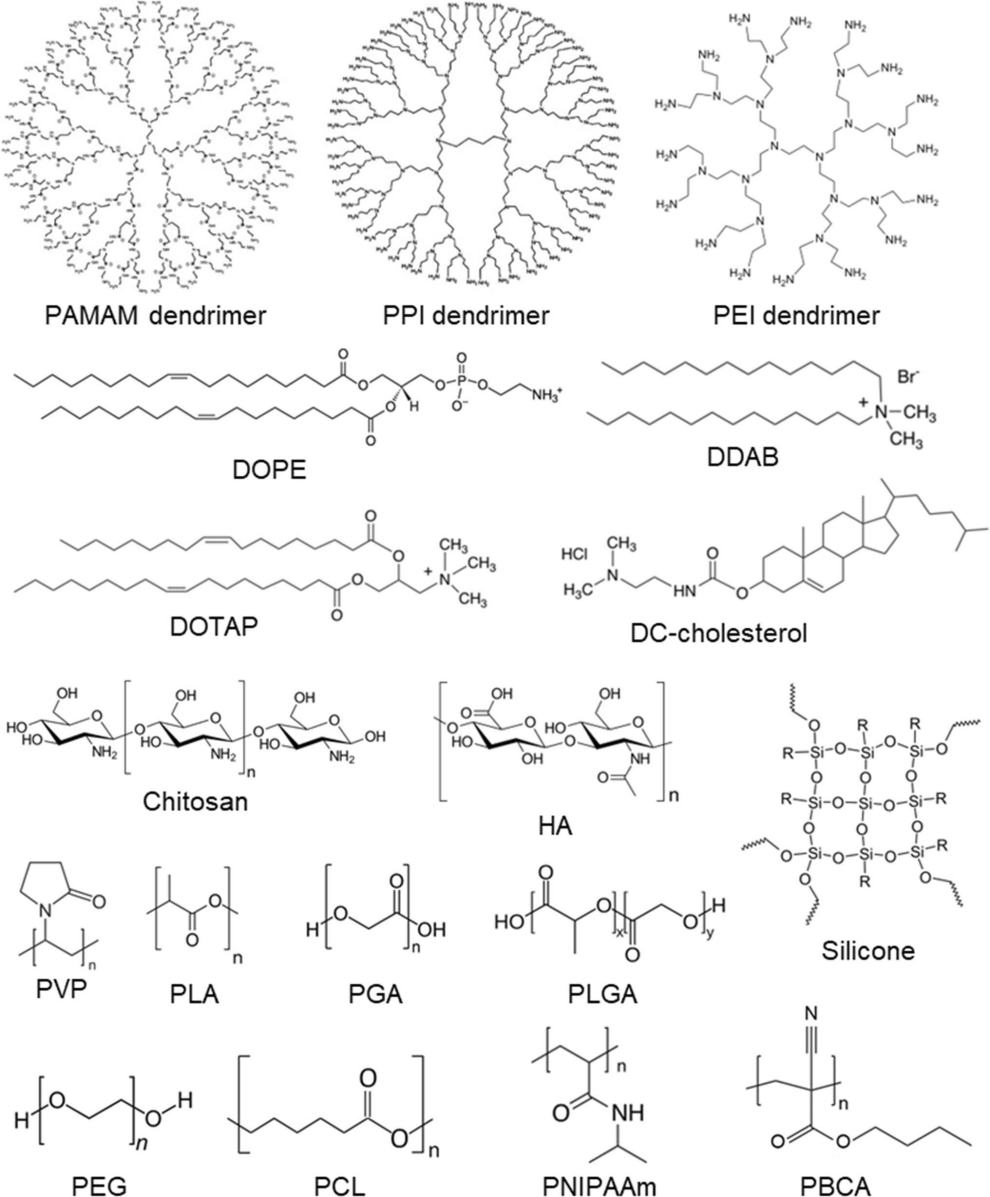
Chemical structures of representative chemicals used for improving drug delivery

Nanomedicine is a medical field that uses nanotechnology for diagnostic or therapeutic purposes. Nanocarriers are fabricated at a nanometer scale allowing encapsulation of different pharmaceutical agents such as small molecule drugs, peptides, proteins, or nucleic acids. They aim at improving drug safety and efficacy by way of encapsulation of hydrophilic or hydrophobic drugs and permitting their sustained or controlled release to avoid the need for frequent intravitreal injections and achieving functionalization for the specific tissue or cell targets. One nanomedicine that received clinical approval is pegaptanib (brand name Macugen, OSI Pharmaceuticals), an anti-VEGF aptamer conjugated with branched polyethylene glycol for the treatment of AMD (Farjo et Ma 2010). Nanocarriers used in retina drug delivery are made of various compositions like organic (polymers, lipids, dendrimers) and inorganic molecules, such as metallic, metal oxides, silicon, silica, carbon, iron oxide, and ceramic (Meza-Rios et al. 2020) (Fig. 5).

**Fig. 6:**
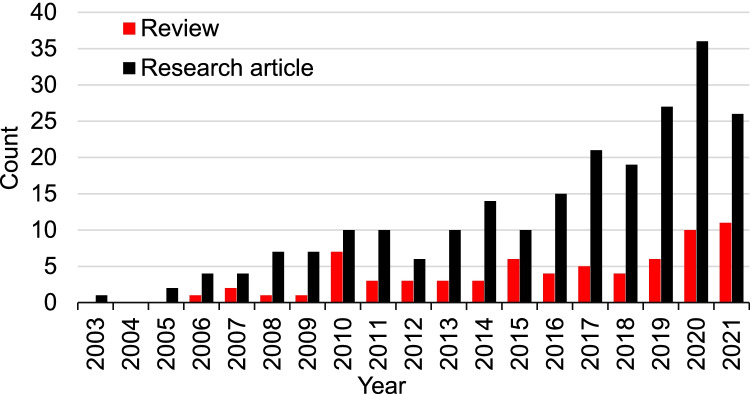
Analysis of literature about retina drug delivery and nanoparticles as of 02/08/2022. The database was PubMed, and keywords were (“retinal drug delivery” AND “nanoparticles”) OR (“retina drug delivery” AND “nanoparticles”) OR (“retinal drug delivery” AND “nanoparticle”) OR (“retina drug delivery” AND “nanoparticle”)

For more information on the relationship between the physicochemical characteristics of the nanoparticles and the therapeutic effect on retinal diseases please check the review by (Jo et al. 2015). Currently, there are 300 articles listed on PubMed (as of 02/08/2022) with the keywords (“retinal drug delivery” AND “nanoparticles”) OR (“retina drug delivery” AND “nanoparticles”) OR (“retinal drug delivery” AND “nanoparticle”) OR (“retina drug delivery” AND “nanoparticle”). The publication year of these articles spans from 2003 to 2021 when the analysis was performed. Approximately 23% of these publications are review papers (70 articles) and the rest are research articles (230 articles). The chronicle of published articles on this topic on PubMed is shown in Fig. 6. It is obvious that the research area of nanoparticles in retinal drug delivery has burgeoned in the 2000s and continues to grow.

#### Liposomes

Liposomes are spherical vesicles, consisting of an inner aqueous layer enclosed by a phospholipid layer. Smaller unilamellar vesicles are composed of one lipid bilayer and have a size of 10 to 100 nm. Larger unilamellar vesicles are also composed of one bilayer but are larger than 100 nm, and multilamellar vesicles are composed of several bilayers and are typically larger than 500 nm. They are biocompatible and biodegradable due to their resemblance in the lipid structure with biological membranes. Moreover, they can encapsulate hydrophobic and hydrophilic drugs (Bozzuto and Molinari 2015).

Liposome-based treatments were the first nanomedicine to receive FDA approval in 1995, and since then more commercially available products became available, such as Visudyne (verteporfin for injection, NDC 0187–5600-15, Bausch and Lomb), Doxil (liposomal doxorubicin, NDC 0338–0063, Baxter Pharma), Novasome, and Nyotran (liposomal nystatin, NDC 0093–0983-01, Aronex Pharmaceuticals Inc.). Visudyne, for example, is a light-activated liposomal formulation of verteporfin for subfoveal choroidal neovascularization in AMD. Another commercial liposome formulation is “Tears again”™ (mineral oil, white petrolatum ointment, NDC 54,799–906, Ocusoft, Inc.), a liposome formulation of mineral oil and white petrolatum for dry eye treatment. Tacrolimus-loaded liposomes (an immunosuppressive drug) are yet another formulation that is intravitreally injected to treat autoimmune uveoretinitis. It remains in the vitreous body for 14 days to reduce intraocular inflammation to prevent disease progression. Also, topical administration of triamcinolone acetonide (TA)-loaded liposomes was tested as an alternative for corticosteroid intravitreal injection for the treatment of macular edema. Liposomes have successfully reached the rabbit retina, and efficacy was confirmed in patients suffering from refractory macular edema. The treatment lasts 90 days and can achieve improvement in visual acuity and central foveal thickness with no side effects (Crommelin et al. 2020). Gu et al. used multifunctional dexamethasone salt-loaded liposomes for topical administration, reaching therapeutic concentration in the choroid and retina within 2 h. This shows that liposomes can be potentially effective carriers for drug delivery to the posterior eye chamber (Gu et al. 2019). Gross et al. studied paclitaxel-loaded cationic liposomes to counteract choroidal neovascularization after intravenous injections (Gross et al. 2013), and Wong et al. used latanoprost-loaded liposomes to reduce IOP after a single subconjunctival injection. Latanoprost was released over 90 days in rabbits’ eyes, with IOP reduction almost twice that of daily topical administration of Latanoprost (Wong et al. 2014a, b). However, it should be recognized that some of the new drug delivery methods carry some risks as well. Bochot et al. observed aggregation of liposomes during storage which can lead to poor stability and blurred vision (Bochot and Fattal 2012). Furthermore, cationic liposomes run the risk to provoke an inflammatory response (Lv et al. 2006). Finally, because of the number of excipients needed during production such as preservatives and fillers, and the complicated fabrication method, liposomes have the general drawback of low physical stability, a major hurdle for well-controlled and sustaining drug release (Bachu et al. 2018).

#### Dendrimers

Dendrimers are water soluble, branched three-dimensional nanostructures made of polymers. Generally, their sizes range from 1 to 100 nm, and they can accommodate hydrophilic and hydrophobic drugs and can be easily functionalized. They can have neutral, negative, or positive functional groups at the terminal of their branches (Kalomiraki et al. 2016; Kokaz et al. 2022). Topical administration of brimonidine tartrate-loaded dendrimer nanofibers was found to be more efficient than conventional eye drops in a rat glaucoma model (Lancina et al. 2017). Carboplatin-loaded poly(amidoamine) (PAMAM) dendrimers were tested in a mouse model of retinoblastoma where they crossed the sclera and provided a sustained suppressive effect on tumor vasculature after subconjunctival injection for 22 days (Kang et al. 2009). Poly (propylene imine) dendrimers loaded with acetazolamide were investigated in albino male rabbits to reduce the IOP to achieve an antiglaucoma effect (Mishra and Jain 2014). Lezzi et al. tested fluocinolone acetonide (TA) conjugated hydroxy-terminated PAMAM dendrimers for the treatment of neuroinflammation in retinal degeneration. An in vitro release profile showed a sustained release for 90 days. In vivo use of dendrimers was able to halt retinal degeneration while preserving the photoreceptors counts for 4 weeks after intravitreal injection (Iezi et al. 2012).

However, dendrimer formulations have not yet reached the clinical stage of development for eye applications. This could be due to their high toxicity profile which might happen when positively charged dendrimers interact with the negative charge of biological membranes, leading to membrane disruption, erosion, apoptosis, and subsequent necrosis. Currently, efforts are underway to reduce the toxicity of acetylation, peptide conjugation, and PEGylation (Albertazzi et al. 2013).

#### Micelles

The typical size range of micelles is 10–100 nm. They are composed of monolayers of amphiphilic molecules that can self-assemble. The amphiphilic molecules can be surfactants, polymers, or other small molecules in nature. When formed above the critical micellar concentration, they can form a hydrophobic core and a hydrophilic shell in an aqueous environment. Depending on the amphiphilic molecule and the solvent used, a different micellar structure can be obtained such as standard, reverse, and unimolecular micelles. Micelles have the advantage of low probability of aggregation and increased circulation time as they are not recognized by the liver macrophages (Cholkar et al. 2012; Patel et al. 2013).

Different studies demonstrated efficient encapsulation and enhanced pharmacokinetics of hydrophobic drugs such as cyclosporine-A, voclosporin, and curcumin for the treatment of posterior eye diseases (Velagaleti et al. 2010; Alshamrani et al. 2019; Mandal et al. 2019). Others tested an optimized aqueous micellar (12 nm) solution of resolving lipid prodrug in a rabbit model after topical administration. Micelles were able to reach the retina again with no sign of retinal damage after multiple doses (Vadlapudi et al. 2014). Ideta et al. reported the treatment of CNV with fluorescein isothiocyanate-labeled poly-L-lysine loaded polyion complex micelles by intravenous injection. Their micelles were able to circulate for 168 h in the body with accumulation in choroidal neovascularization (CNV)-induced lesions (Ideta et al. 2004). Hence, micelles can reduce drug toxicity and drug degradation, thus improving the retinal bioavailability of hydrophobic drugs (Bisht et al. 2018).

Another kind of micelles is nanotubes. For example, Panda et al. studied dipeptide phenylalanine-α, and β-hydroxyphenylalanine nanotubes (Panda et al. 2013) to deliver pazopanib with 25% efficiency and no toxicity in retinal cells after intravitreal injection. Pazopanib was found in vitreous humor, retina, and choroid RPE at higher concentrations for 15 days. These results suggest that nanotubes can also serve as delivery systems to achieve sustained higher drug concentrations in ocular tissues.

#### Organic nanoparticles

Different synthetic biodegradable polymers have been studied for ocular drug delivery. They include polyvinyl-pyrrolidone (PVP), polylactic acid (PLA), poly(lactic-co-glycolic acid) (PLGA), poly(n-butyl cyanoacrylate) (PBCA), polyglycolide (PGA), and polycaprolactone (PCL). They have different advantages of being able to provide controlled and sustained drug release which has greater stability than liposomes and the ability to incorporate hydrophilic and hydrophobic drugs. Though their main advantage is good biodegradability, they have specific drawbacks such as scaling-up problems and insufficient toxicological assessments in the literature (Lahkar and Das 2019; Tsai et al. 2018). Natural polymers such as gelatin and chitosan were reviewed by (Tsai et al. 2018).

##### Polyvinylpyrrolidone

**Fig. 7:**
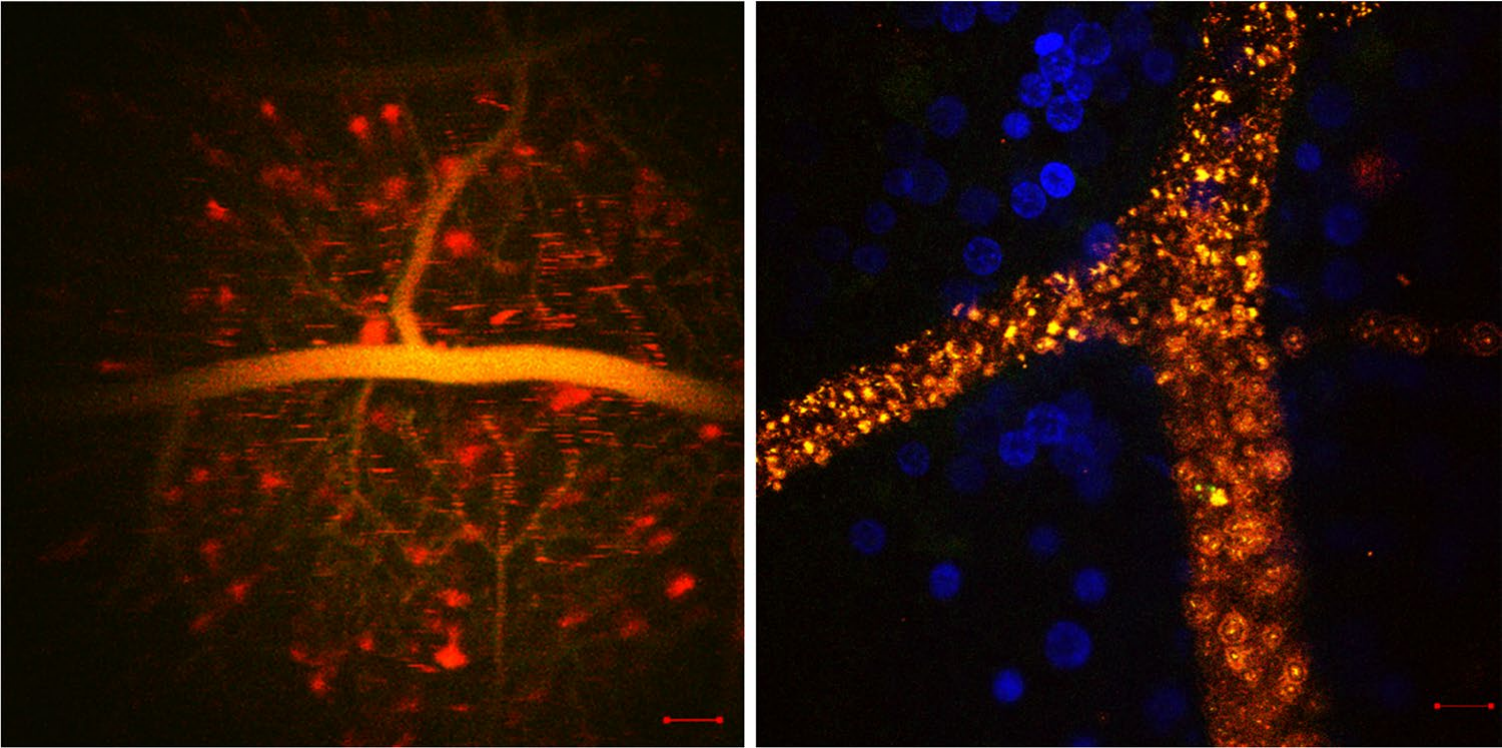
In vivo imaging of Poly-vinyl-pyrrolidone nanoparticles, 5 X. Dil (1,1'-Dioctadecyl-3,3,3',3'-Tetramethylindocarbocyanine Perchlorate) fluorescence in vessels and diffused staining of Dil + Carboxyfluorescein succinimidyl ester (CFSE) in retinal tissue (Left). Scale bar: 200 µm. Ex-vivo imaging of PVP NPs, 50 X, retina wholemount double labelling, blood vessel in green + red (merged signals of Dil and CFSE), Nuclei in blue (Hoechst33342) showing blood cells staining. Scale bar: 20 µm

Polyvinylpyrrolidone (PVP) is a biodegradable polymer used previously for vitreoretinal drug delivery. PVP hydrogels showed great retention at injection sites for several weeks. Some studies showed the potential of cross-linked PVP polymer as an artificial vitreous substance, and they can serve as scaffolds for lens regeneration and delivery system for antiglaucoma drugs providing 300 days of controlled release (Robinson et al. 2018; Bruining et al. 1999; Hong et al. 1996; Gupta 2016). Recently, we were able to deliver small hydrophobic molecules using PVP nanoparticles (PVP NPs) which can cross the BRB after intravenous injection. PVP NPs, when conjugated with the hydrophobic fluorescence marker, Carboxyfluorescein-succinimidyl-ester (CFSE), can be used to image the transport of PVP NPs into retinal tissue. PVP NPs were loaded with hydrophobic fluorescent markers (Dil) as a surrogate for hydrophobic drugs and injected intravenously into the tail vein of rats. PVP-Dil-CFSE NPs had a particle size of 344 nm and a negative zeta potential of -15 mv (Tawfik et al. 2021a, b). We observed a substantial internalization of the modified NPs into blood cells which were revealed by ex vivo whole-mounted retina imaging. This “camouflage” feature shows the potential of biomimetic NPs to deliver drugs while linking synthetic materials with natural compounds (Fig. 7). However, other findings indicated that PVP-based hydrogels can cause a hazy cornea, intravitreal opacity, and inflammation which are known risks (Colthurst et al. 2000).

##### Polylactide, polyglycolide, and their copolymers polylactide co-glycolide

Both polylactide (PLA) and polyglycolide (PGA) are promising retina drug delivery systems, but PGA alone is susceptible to hydrolysis. Polylactide co-glycolide (PLGA) is FDA approved and the most widely used polymer in ophthalmic research for drug delivery (Hyon 2000; Yasukawa 2006). PLA NPs loaded with fluorochrome were injected intravitreally in rats’ eyes, where it was retained in the retina for at least 24 h after injection. Fluorochromes were detected in the RGCs up to 4 months after the injection (Bourges et al. 2003). PLA NPs loaded with betamethasone phosphate were injected intravenously in a rat model of autoimmune uveoretinitis (Sakai et al. 2006). This system showed an effective control of the inflammation in the eye. PLGA-chitosan nanoparticles were able to load and deliver an expression plasmid of Kringle 5 (K5, fragment of plasminogen with antiangiogenic effect) to the retina after intravitreal injection in a rat model. The plasminogen K5 was found in the retina more than two weeks after injection. Moreover, inflammatory cytokines were inhibited as early as three days after injection (Park et al. 2009). Salama et al. loaded brinzolamide into PLGA NPs for the management of glaucoma. After a subconjunctival injection to the eye of albino rabbits, the drug showed sustained release up to 10 days after a single injection while reducing IOP (Salama et al. 2017). Also in rabbits, following intravitreal injection, dexamethasone-loaded PLGA NPs exhibited a sustained release for 50 days and 50% of dexamethasone levels were maintained in the vitreous for 30 days versus 7 days for dexamethasone solution (Zhang et al.^2009^). The targeting effect of PLGA NPs functionalized with transferrin was investigated by intravenous injection in a rat model to target an anti-VEGF interceptor plasmid to choroidal neovascularization lesions. The transferrin conjugation increased the interceptor gene expression in retinal vascular endothelial and RPE when compared to non-functionalized ones (Singh et al. 2009).

In a recent study from our lab, PLGA NPs were loaded either with lipophilic dye 1,1'-Dioctadecyl-3,3,3',3'-Tetramethylindocarbocyanine Perchlorate (DiI) or hydrophilic dye Rhodamine 123 (Rho123) and then coated with poloxamer 188 (P188). Particles had a negative potential and narrow size distribution with an average size of around 130 nm. After intravenous injection in a rat model, DiI signals were detectable for > 90 min in retinal blood vessels. In contrast, Rho123 signals mostly disappeared after 15 min. This showed that the properties of the PLGA carrier-cargo system in the blood circulation of the retina might be strongly influenced by a combination of factors, including the individual properties of loaded compounds and the native blood environment (Zhang et al. 2020a, b).

##### Polycaprolactone

Polycaprolactone (PCL) is a polyester produced from ε-caprolactone through polymerization. It was shown that copolymerizing PCL with PLA or PGA can induce changes in permeability and crystallinity (Yin et al. 2010). Dexamethasone-loaded PCL implant could provide a therapeutic concentration of dexamethasone for 1 year in the rabbit eye (Fialho et al. 2008). Cyclosporine when loaded in PCL nanoparticles showed 10- to 15-fold higher bioavailability than cyclosporine solution in castor oil (Yenice et al. 2008). The PEG-PCL-PEG triblock copolymer was biocompatible with ocular tissues, which makes it a potential nanocarrier for retinal drug delivery (Yin et al. 2010).

##### Poly-butyl-cyanoacrylate

The first polymeric NPs developed for ocular applications were made from poly-butyl-cyanoacrylate (PBCA) (Li et al. 1986). Cyanoacrylates have been widely used in drug delivery because of their favorable properties such as stability, biodegradability, biocompatibility, and targetability (Leggat et al. 2007; Voigt et al. 2014a, b; Vote and Elder 2000). Moreover, polymeric PBCA NPs have been investigated intensively because they could cross the blood–brain barrier (BBB) after being coated with polysorbate 80 (Wilson 2009; Ramge et al. 2000). This was experimentally proven in our lab, by encapsulating the Leu-enkephalin dalargin which normally does not penetrate the BBB when given intravenously. When injected into mice, dalargin-PBCA-NPs coated with polysorbate 80 induced an analgesic effect. Moreover, the intravenous injection of dalargin alone at various doses or dal-PBCA-NPs without the polysorbate 80 was not able to induce an analgesic activity (Schroeder et al. 1998). Furthermore, our lab was the first to investigate different purpose-built PBCA NPs which varied in surfactants, size, and zeta potential. As we showed, the surface coating was a crucial step for BBB passage. This was achieved using our unique In vivo* Confocal Neuroimaging* (ICON) system, which allows live imaging of NPs crossing the BRB, a valid surrogate for the BBB (Voigt et al. 2014b; Sabel et al. 1997; Prilloff et al. 2010; Henrich-Noack et al. 2012). In a different study, we found that decreasing the average zeta size from 272 to 172 nm reduced the BRB passage of PBCA-NPs substantially. Also varying the zeta potential revealed that 0 mV and 15 mV were less effective than 5 mV in terms of facilitating the BRB passage. In sum, PBCA NPs with a larger size and medium surface charge were more likely to accumulate in retinal tissue. As they are easily detected in RGCs, the NPs have an excellent potential to serve as a retinal drug delivery system, and they have a good and well-characterized safety profile in patient studies (You et al. 2019a, 2019b).

#### Inorganic nanomaterials

Inorganic nanoparticles recently gained more attention in ophthalmology. Popular inorganic nanoparticles in biomedical applications are noble metal nanoparticles (gold, silver, platinum), magnetic nanoparticles, silicon-based nanoparticles, ceramic nanoparticles, and carbon-based nanomaterials (Kakoti et al. 2016). Gold NPs (20 nm) were able to cross the BRB after intravenous injection with no significant cytotoxicity, while particles with 100 nm were not able to cross, showing that the passage was size-dependent (Kim et al. 2009). Giannaccini et al. used magnetic nanoparticles to deliver BDNF/NGF into the retina for neuroprotection. They demonstrated that following intravitreal injection in Xenopus embryos, MNPs localized specifically in the retina for three weeks and this prevented RGC-apoptosis significantly which free neurotrophic factors did not. Moreover, through similar experiments in zebrafish, they demonstrated that the targeting of RPE by the nanoparticles is not specific to the Xenopus species (Giannaccini et al. 2017). In another study (Kyosseva and McGinnis 2015), cerium oxide nanoparticles were able to down-regulate caspase-induced apoptosis in the retina of mouse models of AMD and inhibited retinal degeneration after intravitreal injection. Kong et al. showed the potential use of cerium oxide nanoparticles for retina drug delivery, they injected C57BL/6 J mice (retinal degeneration model) with nanoceria, and they found that these nanoparticles protected retinal cells from ROS-induced damage by acting as an efficient antioxidant (Kong et al. 2011).

Finally, Wu et al. reported a slippery micro-propeller that can penetrate the vitreous humor and reach the retina under the wireless actuation of an external magnetic field. The micro-propeller consisted of a helical microstructure with a body of silica as the structural element and nickel or iron as the magnetic segment. The surface was coated with a perfluorocarbon liquid layer to minimize adhesion between the micro-propeller and its surrounding vitreous humor. This system allowed a rapid delivery of concentrated cargo to a defined region at the posterior pole of the eye (Wu et al. 2018). Noteworthy, the micro-propeller can be noninvasively monitored with OCT and in this manner, the location of delivery can be adjusted under imaging guidance. Finally, a considerable issue for inorganic nanoparticles is their poor or absent biodegradation or clearance in vivo, with potentially harmful effects (Jo et al. 2011). In 2021, titanium dioxide NPs were reported to impair the inner blood-retinal barrier function after intravitreal injection which emphasizes the need that cytotoxicity evaluation of inorganic nanoparticles must be carefully considered (Chan et al. 2021).

### Ocular inserts

#### Inserts

Ocular inserts are sterile, drug-loaded microdevices placed in or around the eye for sustained release of therapeutic drugs over a prolonged period. Based on their physical and chemical properties, the inserts are classified as insoluble, soluble, or bio-erodible variants. The inserts increase the ocular surface contact time of the drug lasting between hours to a few days, thereby increasing bioavailability due to a lowered exposure time to tear turnover (Thakur and Swami 2012). The usage of a synthetic bio-soluble matrix in the conjunctival cul-de-sac can also be used to extend the contact time of pilocarpine with the corneal tear film to achieve improved IOP control for the duration of at least 32 h post-insertion (Bensinger et al. 1976).

#### ForSight VISION5

ForSight VISION5 is a non-invasive ocular ring-shaped drug delivery system intended for topical drug application to the eye and is intended as an alternative to eye drops for glaucoma, dry eye, and allergy. The insert contains 13 mg of bimatoprost mixed with silicone matrix without any preservatives and is positioned above polypropylene support (thickness: 1 mm; diameter 24–29 mm). The release kinetics of the drug depends on various factors such as drug concentration gradient, drug molecular diffusivity through the matrix structure, and the surface area of the matrix. The bimatoprost ring provides the release of the drug in a declining manner, with a higher drug release at day 0 (insertion) than at day 180 (removal period). Bimatoprost was a model drug; other prostaglandin like-drugs, latanoprost, or travoprost, can also be loaded into and released by the silicone matrix as well which, for bimatoprost, provides sustained release of up to 6 months. The advantage of the ring is that it is well-retained, noninvasive, and well accepted by patients. About 90% of the patient population was happy while wearing a blank (non-medicated) ring, and excellent retention was found in both eyes for about 6 months during Phase I clinical studies. First Phase II trial results showed a sustained reduction of intraocular pressure (IOP) of 4–6 mmHg (or ~ 20% IOP reduction) for 6 months, high retention, and an excellent safety profile. After the successful trials in Phase II studies, it is now going to enter Phase III (Brandt et al. 2016; Lee et al. 2010).

#### AP-PCL inserts

Alkoxylphenacyl-based polycarbonate copolymers in combination with polycaprolactone (AP-PCL) were examined for sustained delivery of brimonidine tartrate for up to 3 months. The major disadvantage is that it must be administered via the subconjunctival route, requiring invasive surgery. Hence, there is a possibility of subconjunctival migration, infection, and the need for an operating room procedure for the insertion and removal of the device (Manickavasagam et al. 2016).

### Implants

Implants have been frequently investigated to treat retinal diseases. For example, *Ozurdex* (NDC 0023–3348) from Allergan is a dexamethasone implant for macular edema treatment. *Durasert* drug delivery system (pSivida Corp) delivers drugs at various predetermined time points depending on the implant design. The drug release ranges from days to years. *Durasert* consists of a drug core with surrounding polymer layers where the drug release is a function of the polymer layer permeability (Dagnelie 2012; Lloyd et al. 2001; Yasin et al. 2014). *Vitrasert* is the first intravitreal drug delivery system loaded with an antiviral drug (ganciclovir) for the treatment of cytomegalovirus (CMV) retinitis. It utilizes the Durasert technology system and releases the active drug through a small opening in the insert for 6–8 months (Chang and Dunn 2005). *Retisert* intravitreal implant (manufactured by Bausch + Lomb) is a steroid-eluting device implanted surgically in the vitreous humor (Kempen et al. 2011). It releases fluocinolone acetonide up to three years into the vitreous humor and has received fast orphan drug US FDA approval for treatment of posterior uveitis. Posterior uveitis—also called choroiditis—is an inflammation of the choroid capillaries. This can lead to damage to the optic nerve and permanent loss of vision. *Retisert* contains a fluocinolone acetonide tablet encapsulated within a silicone elastomer cup containing an orifice made with a polyvinyl alcohol membrane (Haghjou et al. 2011). *Iluvien* (fluocinolone acetonide intravitreal implant, NDC 68,611–190-02, Alimera Sciences, Inc.) is the most recent US FDA-approved intravitreal injectable insert for the treatment of DME. Multicenter, randomized clinical trials demonstrate that both low and high doses of *Iluvien* resulted in a significant visual improvement with lower side effects. The onset of treatment was very rapid. Patients suffering from DME for more than 3 years had received almost twice the treatment effect as compared to the control group. *Iluvien* is being evaluated in phase II clinical trials for its efficacy in dry AMD (NCT00695318), wet AMD (NCT00605423), and macular edema secondary to RVO (retinal vein occlusion), as compared to Lucentis (ranibizumab, NDC 50,242–080, Genentech, Inc.) treatment (NCT00770770) (Cunha-Vaz et al. 2014; Campochiaro et al. 2012). I-vation TA (SurModics Inc.) is also an intravitreal drug delivery implant for triamcinolone acetonide (TA). It is a titanium helical coil implant coated with TA in a non-biodegradable polymer. Preclinical experiments suggested that I-vation TA can sustain TA release in vivo for up to two years. A phase I safety and efficacy study was conducted on 31 patients with DME after implantation. The implant was well tolerated as indicated by a minimal rise in IOP (Dugel et al. 2009).

### Hydrogels

Hydrogel is a porous water-soluble polymer with a high-water content. It can be formed into particles, films, coatings, or bulky solids. Hydrogels are attractive for drug delivery applications because their physical properties like porosity, swelling ratio, and degradability are highly tunable. Hydrogels are usually highly biocompatible, primarily because of their high-water content and mechanical properties resembling that of extracellular matrix (ECM) (Liu et al. 2016). Drug release from the gel matrix is typically controlled by diffusion through the cross-linked polymer network, which often results in faster drug release than water-insoluble polymer formulations.

The highly porous structure of hydrogels could lead to low tensile strength and instability upon injection. However, optimized hydrogel formulations that take advantage of copolymer additives could increase the cross-linking density and therefore alter the physical properties. Changing the temperature, pH, ionic strength, and shear stress could also help form an injectable solid matrix. In a study by Yu et al. intravitreal injection of hydrogel was able to retain therapeutic concentration of bevacizumab in rabbit eyes vitreous (∼50 μg/mL) even 6 months after injection which was estimated by the simulation to be 10^7^ times higher than the concentration after bolus injections (Yu et al. 2015).

Hydrogel-based formulations have been widely investigated for ocular inserts via subconjunctival injection, topical eyedrops, and combination systems such as hydrogel-embedded liposomes (Bennett 2016).

#### Composite drug delivery systems

Healthy eyes can clear microparticles within 50 days and vitrectomised eyes can clear microparticles within 14 days. To limit particle movement in the eye, injectable hydrogels can be good candidates as the second carrier for nano- and microparticles to provide localized and extended drug release. This composite drug delivery systems (DDS), a mixture of microparticles/nanoparticles and hydrogel, also offers advantages over both particles and hydrogels alone by further extending release and reducing initial bursts. In addition, both proteins and small molecules can be encapsulated into particles and hydrogels in a variety of ways to enhance delivery potential.

One system containing colloidal nanocarriers in a chitosan-based gel was able to sustain IOP reduction for 40 days in a rabbit glaucoma model (Hsiao et al. 2014). Recently, this strategy was validated (Osswald and Kang-Mieler 2016) by combining injectable PNIPAAm-based thermo-responsive hydrogel with PLGA microspheres to create a microsphere-hydrogel composite DDS. The DDS was able to encapsulate ranibizumab or aflibercept and release them in a controlled manner for ~ 200 days. In vitro bioactivity during release and in vivo efficacy in laser-induced CNV rodent models have been established as well (Hirani et al. 2016; Joseph and Venkatraman 2017).

### Contact lenses

Since the widespread adoption of soft, gas-permeable materials in the 1980s, contact lenses are a convenient option for vision correction, with approx. 30 million users in the USA as reported by FDA in 2010. Contact lenses are now being investigated for their potential to serve as drug delivery systems (Gupta and Aqil 2012). The simplest method for increasing the residence time of the drug on the cornea is adsorption of the drug onto traditional contact lenses which offers a potential replacement for eyedrop administration. However, these systems cannot sustain drug release beyond 1 day and require high levels of the drug to ensure adequate loading, with the risk of unwanted release bursts (Fonn 2007).

To overcome this hurdle, other groups have investigated novel materials for contact lens-based drug delivery. One such system uses timolol-loaded nanoparticles within a silicone hydrogel contact lens, which reduced IOP for 192 h in a rabbit model. Another system used molecularly imprinted lenses which showed a significant reduction in IOP compared to high-dose eye drop therapy. Additionally, surfactant-coated contact lenses increased the release duration of dexamethasone 21-disodium phosphate from about 2 to 50 h. Moreover, contact lenses have a high surface charge which helps the adsorption of the ionic compounds onto their surface with high affinity, thus decreasing the transport rate and extending the release time.

The main disadvantage of contact lenses for drug delivery is the incidence of low patient compliance, which is as low as 53% for replacement of lenses and 45% for proper handling. This represents a major drawback for lens-based drug delivery applications, as lenses would need to be changed at the appropriate intervals to ensure that therapeutic drug levels are being delivered. Moreover, inadequate handling and low compliance could lead to additional complications like contact lens-related dry eye and ocular surface infections (Fedorchak 2016; Dumbleton 2002; Dixon et al. 2015; Hsu 2015; Ciolino et al. 2014). This could be improved by using dissolvable lenses made of the natural protein collagen, which is the major component of the cornea and sclera.

#### Collagen shield

The collagen shield was first used as a postoperative corneal bandage. Thereafter, wafer-shaped collagen inserts were examined for drug delivery in rabbit eyes when loaded with gentamicin. A high concentration was found in the tear film and tissues as compared with subconjunctival injection, eye drops, or ointment. Regarding hydrophilic drugs, they were loaded into the collagen matrix by soaking a dry shield in the drug solution. Hydrophobic drugs are directly added to the shield during the manufacturing process. As a hydrogel-based lens, the collagen shield also benefits from composition with nanoparticles. Crosslinked collagen shields consisting of nanoparticles made of titanium dioxide (TiO2), zinc oxide (ZnO), and polyvinylpyrrolidone capped zinc oxide (ZnO/PVP) were developed for controlled delivery of pilocarpine hydrochloride in glaucoma patients over time intervals. In the case of the latter, a sustained release of pilocarpine hydrochloride was achieved for 14 days (Tannebaum 1995; Bloomfield et al. 1978; Reidy et al. 1990).

### Microneedles

The large surface area of the sclera (~ 95% of the total ocular surface area of the eye) offers the possibility of delivering neuroprotective agents, antioxidants, or antiangiogenic agents to specific locations of the retina via transscleral absorption. A major challenge of transscleral delivery is that with high drug clearance mechanisms and static, dynamic, and metabolic barriers, an effective drug concentration within the eye may not be readily achieved. Microneedles enable minimally invasive delivery of free or encapsulated drugs. Clearside Biomedical Inc. developed a microneedle and injector that administers a suprachoroidal injection of corticosteroid triamcinolone acetonide (CLS-TA) (Patel et al. 2011). The injector allows consistent insertion of microneedles into the suprachoroidal space, reducing the risks commonly associated with intravitreal injections, such as retinal damage. Due to their small surface area, microneedles only enable the delivery of small molecules and cannot always deliver a desired therapeutic dose. A phase 3 study showed promising results where 46.9% of patients treated with the CLS-TA microneedle had an increase in visual acuity from baseline as compared to only 15.6% of the control patients. As for safety, 11.5% of treated patients had increased intraocular pressure (IOP) but the control patients did not have any increases, which indicates a higher risk of using the microneedle. They also conducted a Phase 2 clinical trial (TYBEE) for a combination therapy of suprachoroidal CLS-TA with intravitreal injections of aflibercept in patients with macular edema (DME) over a 6-months evaluation period. The goal was to deliver a combination of TA and anti-VEGF to reduce the number of microneedles retreatments. Patients received either quarterly treatment of CLS-TA and intravitreal aflibercept (months 0 and 3) or four monthly treatments of intravitreal aflibercept with a sham suprachoroidal procedure (months 0, 1, 2, and 3). If needed, either group received intravitreal aflibercept at months 4 and 5. The trial met its primary endpoint of mean improvement in best corrected visual acuity (BCVA) from baseline to 6 months using the Early Treatment of Diabetic Retinopathy Trial (ETDRS) scale. Patients gained on average 12.3 ETDRS letters compared to 13.5 ETDRS letters in the control group. The study also met its secondary endpoint with a mean reduction from baseline of 208 µm in central subfield thickness at 6 months (Jiang et al. 2007; Yeh et al. 2020; Identifier NCT02595398).

### Microelectromechanical system

The microelectromechanical system (MEMS) is based on the principle of bubble generation using electrolysis to release the loaded drug out of a reservoir. It is still in the preclinical study phase and opens the possibility to load drugs multiple times. The procedure resembles the implantation of a glaucoma aqueous drainage device. It enables practitioners to fill the drug without the need for an invasive procedure. A single dose is sufficient for usage for 3 to 4 months, and the rate of drug release can be controlled using electrolysis. The only drawback: it requires a minimally invasive procedure for implantation in the eye which will impose associated side effects (Saati et al. 2009; Singh et al. 2020).

### Encapsulated cell technology

Renexus (NT-501, Neurotech Pharmaceuticals, Inc.) is an Encapsulated Cell Technology (ECT) for ocular implantation of human RPE transfected with a plasmid encoding ciliary neurotrophic factor (CNTF). Renexus (NT-501) achieved a Phase III investigation for dry AMD, glaucoma, and retinitis pigmentosa (NCT03316300, active, not recruiting). The implant consists of a hollow tube capsule consisting of a polymeric matrix that can be loaded with genetically modified cells. Various biocompatible polymers like collagen and hyaluronic acid hydrogel are utilized for forming the matrix of ECT. The implant capsule is semipermeable, allowing the diffusion of proteins across the membrane but inhibiting the entry of immune cells. The genetically modified cells in the matrix draw nutrients from the surrounding tissue after implantation. The encapsulated cell technology is implanted in the pars plana and affixed to the sclera (Zhang et al. 2011, 2012; Wong et al. 2017).

## Gene therapy

The eye possesses important features that make it very suitable for gene therapy: well-defined anatomy, relative immune privilege, accessibility, ease of diagnosis, and in the same subject one eye can be used as the experimental target and the other one as a control. The progress in gene therapy holds considerable promise for the management of ophthalmic conditions, and ocular gene therapy has been extensively explored in recent years as a therapeutic avenue to target diseases of the retina, mainly the retinal pigment epithelium (RPE). There are basically two strategies for gene therapy: either restore the function of a nonfunctional or absent protein (gene addition or gene editing) or knock down proteins in order to block their function (gene silencing).

Consistently, effective gene therapy for ocular disease treatment relies on the development of appropriate delivery systems (Solinís et al. 2015). Currently, 47 approved, in progress, or completed clinical trials for ocular gene therapy have been reported according to the Journal of Gene Medicine Clinical Trial (https://a873679.fmphost.com). Viral vectors are the most popular gene delivery systems for ocular diseases with approval of AAV-based therapy. For example, Luxturna (voretigene neparvovec-rzyl, NDC 71,394–415, Spark Therapeutics, Inc.) has been approved by the FDA for the treatment of Leber congenital amaurosis, a retinal disease that can now be treated by gene replacement via a subretinal injection (Bordet and Behar-Cohen 2019). However, in vivo gene expression using viral vectors is still limited by the poor payload, laborious customization, and risks associated with the virus, such as the immune response to viral antigens. Because of this risk-profile limitation, new delivery methods like nanoparticles are needed.

On the other hand, gene silencing through RNA interference was used only in 5 of the 47 studies using direct injections of naked small interfering RNA (siRNA). It is now well established that drug trials of diseases that have a genetic basis are more likely to succeed. In posterior segment diseases, it can be used as a restorative or neuroprotective tool depending on when during the disease course it is applied (before or after pathology) and which gene is being targeted (Guzman‐Aranguez et al. 2013). In a recent study, Leber congenital amaurosis was treated with the antisense oligonucleotide (AON) sepofarsen. One patient who was part of a larger cohort was investigated for 15 months after a single intravitreal sepofarsen injection. Measures of visual function and retinal structure reached a substantial efficacy peak near 3 months after injection. At 15 months, there was sustained efficacy, even though there was evidence of reduction from peak response (Cideciyan et al. 2021). Table 4 summarizes the progress of novel gene therapy and delivery approaches.

**Table 4:** Summary table on gene therapy approaches for retinal diseases

Gene therapies	Applications in retinal diseases treatment and concerns
*A. Viral vectors*	
AAV-based therapy	Luxturna: gene addition via a subretinal injection to treat Leber congenital amaurosis, a retinal disease. Concern: similar AAV2-RPE65 vectors indicated progressing retinal degeneration despite gene augmentation
	• AAV2 vectors transduce RPE and photoreceptors cells upon subretinal administration • AAV2 vectors transduce ganglion cells upon intravitreal injection
	• ADVM-022 (Adverum Biotechnologies Inc.) for sustained intraocular expression of aflibercept
	• AAV packaged with a single 8.9 kb transgene cassette express ABCA4 protein in the mouse retina • AAV expresses REPE65 transgene after subretinal injection in diseased RPE cells of the RPE65 deficient mouse • A novel AAV8 vector (RGX-314) encoding a soluble anti-VEGF monoclonal antibody fragment for wet AMD treatment
	• Drawbacks: poor payload, laborious customization, and risks associated with the virus—such as the immune response to viral antigens
Lentivirus	• Lentiviral vectors can transduce photoreceptors in the newborn mouse retina but have a much lower capacity than AAV vectors to transduce the adult photoreceptor
	• Lentiviral vectors can transduce retinal cells with different genes related to retinal dystrophies • RetinoStat via subretinal injection can express the angiostatic proteins for the treatment of wet AMD
siRNA	• siRNA therapy is well tolerated in patients with neovascular AMD and may improve visual acuity • The first siRNA therapy is approved for the treatment of polyneuropathy in people with hereditary transthyretin-mediated amyloidosis using lipid nanoparticles and intravenous injection
	• Bevasiranib is an intracellular transcriptional inhibitor of VEGF and an anti-angiogenic agent for the treatment of wet AMD
	• AGN211745 reduces the level of VEGFR-1 mRNA to inhibit CNV
	• SYL040012 specifically inhibits the synthesis of ADRB2 and reduces the IOP
	• QPI-1007 protects RGCs in optic neuropathy by RNAi inhibition of caspase-2
Aptamers	• Pegaptanib, an RNA aptamer, has shown significant improvement in the treatment of “back-of-the-eye diseases” • E10030, a DNA aptamer, has been used against platelets-derived growth factor (PDGF) • ARC1905 has been utilized in combination with ranibizumab for the treatment of subfoveal CNV secondary to AMD • Early treatment of miRNA-124 aimed at preventing neuronal damage demonstrated certain anti-inflammatory and neuroprotective effects
*B. Non-viral vectors* Polymeric vectors	• PLGA microspheres could achieve a high and sustained suppressive effect on VEGF gene expression • Nanoparticles with a hydrophobic PLGA/folate core and a hydrophilic PEGylated polymeric lipid shell for drug and gene co-delivery • PLGA nanospheres and microspheres loaded with EGFR TKI4 for intravitreal injection to promote optic nerve regeneration after optic nerve injury • Dual poly-VEGF siRNA formed stable NPs with thiolated glycol chitosan and inhibited VEGF gene expression and tumor growth • Anti-VEGF nanospheres constructed by electronically coating siRNA hydrogel with PEI and hyaluronic acid entered the subretinal space through the vitreous humor and escaped the immune response of TLR3 • HA-modified cationic niosomes showed remarkable transfection efficiency to retina layers • PBCA NPs encapsulated the caspase-3 siRNA and induced an anti-apoptotic effect in the retina
Lipid NPs	• Pegylated liposome-protamine-hyaluronic acid nanocarriers loaded with siRNA targeted against VEGFR1 reduce the area of choroidal neovascularization (CNV)
	• Three cationic liposome formulations (TMAG, DDAB, and DC-cholesterol) delivered reporter genes to the ganglion cells and RPE through subretinal and intravitreal injections
Protein NPs	• Cell-penetrating peptide (CPP) for ocular delivery of siRNA and plasmid DNA to RPE, photoreceptor, and ganglion cells
Dendrimers	• Dendrimers with amine-containing cationic polymers such as poly-L-lysine can deliver anti-VEGF to RPE cells in vitro and inhibit laser-induced CNV after intravitreal injection
	• PEI dendrimers can deliver an shRNA expression plasmid to retinal ganglion cells via intravitreal injection
Gold NPs	• Gold-PEI NPs showed a higher transfection rate than PEI NPs • Gold-PEI NPs can transfect human corneal cells in vitro and in vivo

### RNAs and aptamers

#### Small interfering RNA (siRNA)

Small interfering RNA (siRNA), sometimes referred to as short interfering or silencing RNA, is a double-stranded RNA molecule with 20–25 base pairs that can be used to induce gene silencing in cells. Typically, gene silencing takes place after the transcription stage. The application of siRNA for various posterior segment ocular diseases may be considered a promising approach. siRNA therapy is well tolerated in patients with neovascular AMD and may improve visual acuity. The first siRNA-based therapy has received marketing approval for the treatment of polyneuropathy in people with hereditary transthyretin-mediated amyloidosis using lipid nanoparticles after intravenous injection. It may comprise a new nanotechnology platform with the potential for the use of siRNA as a novel means of systemic administration for gene therapy. Thus, siRNA therapy represents an important new class of therapy against ocular diseases (Jiang et al. 2021; Hu et al. 2020).

##### Bevasiranib

Bevasiranib is a chemically modified naked RNA, an intracellular transcriptional inhibitor of VEGF, and an anti-angiogenic agent for the treatment of wet AMD. Bevasiranib localized to the retina and a relatively high amount of bevasiranib was measured in Bruch’s membrane after IVT injection in rabbit models, indicating that the drug distributed well in the eye and reached its target tissues. Heterologous monkeys with laser-induced CNV were treated with three doses of bevasiranib (70, 150, or 350 µg). Compared with the control, bevasiranib significantly inhibited the growth of new blood vessel areas. The overall reduction of CNV area in the bevasiranib group was greater than 50% of the control group (Dejneka et al. 2008; Tolentino et al. 2004).

##### AGN211745

AGN211745 (formerly Sirna-027) is a chemically modified naked siRNA. The target gene is VEGFR-1, which reduces the level of VEGFR-1 mRNA to significantly inhibit CNV, aiming at wet macular degeneration treatment. Shen et al. used retinal and choroidal CNV mice as models, and IVT injection and periocular injection of AGN211745, respectively. In both approaches, AGN211745 was detected in the retina for 4–5 days, indicating that it can remain in retinal cells for a considerable period, and correspondingly reduce VEGFR-1 mRNA and protein. Kaiser et al. recruited 26 patients with a median age of 82 and CNV caused by AMD where they received a single intravitreal dose of AGN211745. The adjusted mean foveal thickness decreased within 2 weeks after study treatment, and improvement in visual acuity and foveal thickness was observed (Kaiser et al. 2010; Shen et al. 2006).

##### SYL040012

SYL040012 is a chemically modified naked RNA, which specifically inhibits the synthesis of the b2-adrenergic receptor (ADRB2) without affecting the expression of other receptors in the adrenergic family. The target of SYL040012 is ADRB2 mRNA, thus inhibiting the expression of ADRB2 which reduces the level of ADRB2 mRNA and the IOP. Martınez et al. tested the *in vivo* efficacy of SYL040012 in a water load-induced glaucoma rabbit model with target gene expression reduced by approximately 50% in animal models after administration over 4 days, resulting in a significant decrease in IOP. The cynomolgus monkey was tested for safety in a topical ocular instillation over 28 days, and clinical and histopathological examination showed that the drug was safe and well tolerated (Moreno-Montañés et al. 2014; Martínez et al. 2014). Phase 1 clinical trial (NCT00990743) of SYL040012 evaluated the safety, tolerability, and bioavailability of 30 healthy volunteers with intraocular pressures below 21 mmHg. The results indicated that the single and repeated administration of SYL040012 was safe and well tolerated, with no pathological changes in the eye at all times. Phase II clinical trial (NCT01739244) investigated the tolerance and ocular hypotensive effect of SYL040012 in patients with high intraocular pressure or open-angle glaucoma. The drug was well tolerated, and 14.6% of patients had mild reactions (Ocular discomfort and adverse events (AEs) occurrence). SYL040012 caused a significant reduction in IOP at a dose compared to the placebo group or baseline. Four doses of SYL040012 eye drops were evaluated in the phase II clinical trial called SYLTAG, which was used to reduce IOP in patients with open-angle glaucoma (NCT02250612) compared to 0.5% timolol maleate. All treatments decreased IOP for 28 days, and SYL040012 had the highest effect compared with baseline. In terms of toxicity, no grade 3 or 4 adverse events occurred during the 28-day treatment period (Gonzalez V et al. 2014; Gonzalez V et al. 2016).

##### QPI-1007

QPI-1007 is a 19-nucleotide naked RNA duplex targeting caspase-2, which provides neuroprotection by RNAi inhibition of this pro-apoptotic protein, protecting RGCs in optic neuropathy by IVT injection. It is being developed as a neuroprotectant for the treatment of non-arteritic anterior ischemic optic neuropathy and other optic neuropathies such as glaucoma that result in the death of RGCs. Solano et al. administrated QPI-1007 with intravitreal injection in Dutch belted rabbits and found no accumulation after repeated administration at 2 or 4 weeks of dosing in rabbits. QPI-1007 was well tolerated in Dutch-belted rabbits following single or repeated IVT administrations of up to 11 doses over 9 months. Rats were given a single intravenous injection for 28 days. However, both failed to elicit any macroscopic or microscopic changes, suggesting a low risk for systemic toxicity. QPI-1007 has achieved phase 2/3 of clinical trials (NCT02341560) but is currently terminated (an interim analysis did not warrant continuing enrollment) (Titze-de-Almeida et al. 2017; Solano et al. 2014).

#### Aptamers

Aptamers are single-stranded DNA or RNA (ssDNA or ssRNA) oligonucleotide ligands that bind to molecular targets with high affinity and specificity. RNA aptamer (Pegaptanib) has been studied for the treatment of posterior ocular diseases where it showed significant improvement in the treatment of “back-of-the-eye diseases” (Amadio et al. 2016). Another aptamer E10030—a DNA aptamer—has been used against platelets-derived growth factor (PDGF) which stimulates angiogenesis. It is currently in Phase III clinical trial, in combination with anti-VEGF agents for the treatment of AMD. Because inhibition of PDGF escalates the sensitivity of endothelial cells to anti-VEGF agents, combination therapy of anti-VEGF and other growth factors may be beneficial to improve vision (Ishikawa et al. 2015). Another aptamer, ARC1905, has also been utilized in combination with ranibizumab for the treatment of subfoveal CNV secondary to AMD (NCT01940900). Recently, early treatment of miRNA-124 aimed at preventing neuronal damage demonstrated certain anti-inflammatory and neuroprotective effects (Taj et al. 2016).

### Viral vectors

Several viral vectors such as adenovirus, adeno-associated virus (AAV), and lentivirus are extensively investigated in ocular gene therapy. Earlier generations have been optimized using self-complementary AAV or helper-dependent adenovirus, enhancing loading capacity. Moreover, viral vectors are preferred due to the long-lasting gene expression in comparison with nanoparticles which are limited to a few weeks only. However, viral vectors are considered acceptable rather than optimal solutions for nucleic acid delivery due to the potential of mutagenesis, limited loading capacity (5 Kbp for AAV), adverse immune reactivity, and high production cost which consequently leads to unaffordable measures for patients (Luxturna cost 425,000 $ per eye) (Liu et al. 2011a, b; Mak KY et al. 2017; Kaemmerer 2018).

#### Adeno-associated virus

To date, thirteen wild-type adeno-associated virus (AAV) serotypes (AAV1–AAV13) have been isolated Among them, AAV2 is the most commonly used for gene delivery (Srivastava A et al. 2016; Tseng and Agbandje-McKenna 2014). Following subretinal administration, the AAV2 vectors result in the transduction of RPE and photoreceptors cells while intravitreal injection leads to ganglion cell transduction (Stieger et al. 2008). Similar promising results were recently reported by Adverum Biotechnologies Inc, which develops a new candidate, ADVM-022 (AAV2.7m8-aflibercept) to be administered as a single IVT injection. In the non-human primate laser-induced choroidal neovascularization (CNV) model, a single IVT injection of ADVM- 022 allowed for the sustained intraocular expression of aflibercept for up to 16 months with long-term efficacy in preventing the development of Grade IV lesions. The company initiated a Phase I trial in 2018 to assess the safety and tolerability of a single IVT administration of ADVM-022 in anti-VEGF patient responders (NCT03748784) (Grishanin et al. 2019). In 2008, the expression of ABCA4 protein was demonstrated in the mouse retina from a single 8.9 kb transgene cassette packaged into AAV (Allocca et al. 2008). In diseased RPE cells of the RPE65 deficient mouse, scAAV REPE65 transgene expression was detected as early as four days after subretinal injection (Pang et al. 2010). For wet AMD, results are available from a Phase I, open-label dose-escalation trial evaluating the safety and early efficacy of the subretinal injection of a novel AAV8 vector (RGX-314) encoding a soluble anti-VEGF monoclonal antibody fragment (NCT03066258). Dose-dependent protein expression levels were observed at 1 month, with sustained expression demonstrated at 6 months in patients treated at a dose of 6E10 vector genome per eye. For 6 months, most patients treated at that dose had required minimal or no anti-VEGF injections (50% of patients), with the maintenance of central retinal thickness and BCVA assessments versus baseline showing maintenance or improvements in visual acuity. Although trials with Luxturna reported long-term encouraging efficacy data, other studies with similar AAV2-RPE65 vectors indicated progressing retinal degeneration despite gene augmentation (Bordet and Behar-Cohen 2019).

#### Lentivirus

Recombinant lentiviral vectors are also being tested in phase 1/2 of retinal gene therapy, although they are a much less common vehicle than AAV (Auricchio et al. 2017). Studies in mice have shown that subretinal use of lentiviral vectors appears to be well tolerated (Bartholomae et al. 2011). Lentiviral vectors are known to be able to transduce photoreceptors in the newborn mouse retina leading to phenotypic improvements in animal models of IRDs, including those for Stargardt disease and Usher syndrome, a form of retinitis pigmentosa (RP) associated with hearing loss. However, lentivirus vectors appear to have a much lower capacity than AAV vectors to transduce the adult photoreceptor (Kong et al. 2008; Binley et al. 2013; Hashimoto et al. 2007; Trapani et al. 2015). Lentiviral vectors are also able to transduce retinal cells, and they have been studied as vectors for different genes related to retinal dystrophies such as rod photoreceptor cGMP phosphodiesterase beta subunit (PDEbeta), RPE65 or photoreceptor-specific adenosine triphosphate (ATP)-binding cassette transporter (ABCA4) protein (Kong et al. 2008; Binley et al. 2013; Bemelmans et al. 2006; Takahashi et al. 1999). Binley et al. have designed a lentiviral vector, RetinoStat (OXB-201, Oxford Biomedica), which expresses the angiostatic proteins endostatin and angiostatin, to be delivered via a subretinal injection for the treatment of the wet form of age-related macular degeneration (Binley et al. 2012). Among non-viral strategies, physical methods (iontophoresis, electroporation, gene gun, nucleofection) have achieved considerable progress, but gene expression efficiency is still a limitation, and non-viral vectors have gained more attention (Naik et al. 2009; Cai et al. 2008).

### Non-viral vectors

#### Polymeric vectors

PLGA is able to efficiently transfect RPE cells after IVT injection in rats without significant toxicity (Oliveira et al. 2017). Murata et al. invented injectable anti-VEGF PLGA microspheres containing siRNA with arginine or PEI which functions as a siRNA vector that can lead to high siRNA encapsulation and transfection efficiency. The above microspheres have achieved a higher and sustained suppressive effect on VEGF gene expression (Murata et al. 2008). Wang et al. created a kind of NPs that consists of a hydrophobic PLGA/folate core and a hydrophilic PEGylated polymeric lipid shell which successfully functioned as the co-delivery of drugs and genes (Wang et al. 2010). While the intravitreal route is commonly used for the delivery of NPs to the retina, Singh and colleagues explored whether PLGA nanoparticles (NPs) (containing a plasmid that expresses an anti-VEGF peptide) could be specifically delivered to the laser-treated retinas when administered via the intravenous route (Singh et al. 2009). Epidermal growth factor receptor (EGFR) tyrosine kinase inhibitors (TKI) are known to promote the survival of neurons. Robinson et al. fabricated PLGA nanospheres and microspheres loaded with EGFR TKI4 for intravitreal injection to promote optic nerve regeneration after optic nerve injury. Two weeks after injection, they were able to detect fewer microspheres than nanospheres and only nanospheres were detected after 4 weeks. Both particles induced optic nerve regeneration at two weeks but only nanospheres injected into animals showed regeneration after 4 weeks (Robinson et al. 2011).

One study reported that dual poly-VEGF siRNA formed stable NPs with thiolated glycol chitosan via chemical bond formation and charge interaction. NPs were highly accumulated in tumors, resulting in efficient inhibition of VEGF gene expression and tumor growth without serious side effects (Kim et al. 2017). Ryoo et al. produced novel siRNA-based anti-VEGF nanospheres which were constructed by electronically coating siRNA hydrogel with poly(ethylenimine) and hyaluronic acid (HA). It was found that nanospheres entered the subretinal space through the vitreous humor and escaped the immune response of TLR3. The therapeutic effects of nanospheres lasted for 2 weeks after IVT injection, showing high targeting efficiency for the SR space. Other studies reported HA modified cationic niosomes (HA-C-niosomes) composed of DOPE, DOTAP, and HA, showed remarkable transfection efficiency to retina layers, compared with the niosome groups with no HA modification (Ryoo et al. 2017).

**Fig. 8:**
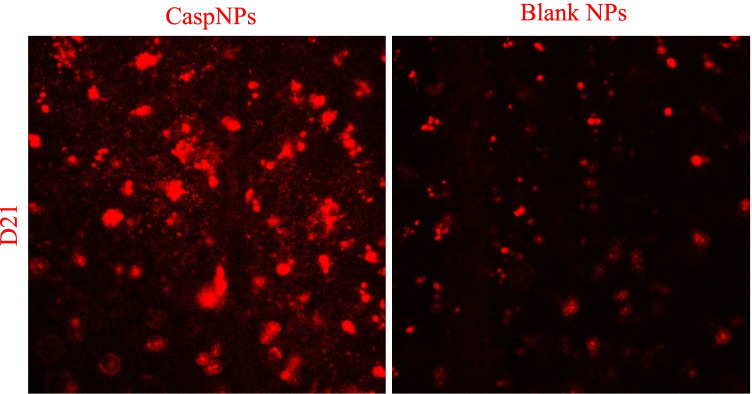
Representative photomicrographs of retinal ganglion cells as labelled by 2 μl microspheres in retinal whole mounts post-optic nerve damage. Day 21 shows more retinal ganglion cells in rats treated with CaspNPs (left) than those treated with blank NPs (right). Scale bar 20 µm

Finally, our lab showed the capability of PBCA NPs to encapsulate the caspase-3 siRNA and induce an anti-apoptotic effect in vitro and in vivo. In vitro*,* PBCA NPs significantly blocked caspase-3 protein expression in C6 cells, and when injected intraocularly in vivo, CaspNPs lowered retinal caspase-3 immunofluorescence by 57.9% in rats with optic nerve crush. Longitudinal, repeated retinal ganglion cell counts using confocal neuroimaging showed that post-traumatic cell loss after intraocular CaspNP injection was only 36.1% versus 63.4% in lesioned controls (Tawfik et al. 2021b) (Fig. 8).

#### Lipid NPs

Liu et al. have successfully demonstrated that 132 nm pegylated liposome-protamine-hyaluronic acid nanocarriers loaded with siRNA targeted against VEGFR1 can not only enhance VEGFR1 knockdown but also accelerate intracellular delivery to human RPE cells over free siRNA in vitro. After intravitreal administration, these nanocarriers were also able to significantly reduce the area of choroidal neovascularization (CNV) in a laser-induced murine CNV model with minimal toxicity, suggesting their suitability for clinical applications (Liu et al. 2011a, b). Lipid combinations such as DC-Chol (cholesterol) have also been used to deliver siRNA successfully and may present opportunities to combine desired features to create novel lipid-based nanocarriers (Zhang et al. 2010).

Masuda et al. were among the first to compare the efficacy of three cationic liposome formulations (TMAG, DDAB, and DC-cholesterol) for gene transfer to rat ocular tissues via different administration routes: topical application, or injection into the anterior chamber, subretinal, and intravitreal injections. The authors showed that subretinal and intravitreal injections of all three liposomes resulted in the expression of the lacz reporter gene in the ganglion cells and RPE, with TMAG liposomes being the most efficient. However, none were successful in transfecting photoreceptor cells (Masuda et al. 1996). Afterwards, Kachi et al. performed an extensive study to evaluate the safety and effectiveness of ocular gene transfer using commercial cationic liposome reagents (lipofectamine 2000 and NeuroPorter). Intravitreal injection of the lipofectamine-packaged plasmid (CMV promoter-driven lacz) induced beta-galactosidase expression only in the ganglion cell layer. On the other hand, subretinal injection of the complex induced high levels of gene expression in photoreceptors and RPE cells within 3 days post-injection. Lipofectamine 2000 was found to be highly toxic to photoreceptor cells as indicated by the thinning of the outer nuclear layer with a decrease in ERG a- and b-wave amplitudes at 14 days post-injection. NeuroPorter on the other hand was non-toxic to the retina, but it was only able to transfect the RPE (Kachi et al. 2005).

#### Protein NPs

Protein-based siRNA delivery involves the formation of “proticles,” where proteins are conjugated (electrostatically or covalently) to siRNA for delivery. For example, albumin protamine-oligonucleotide forms nanocarrier complexes (230–320 nm diameter), which can be safely delivered to cells (Lochmann et al. 2005). Recently, Johnson et al. have developed a novel cell-penetrating peptide (CPP) for ocular delivery of small and large molecules, including siRNA, fluorescent probes, plasmid DNA, and quantum dots to RPE, photoreceptor, and ganglion cells in vitro and in vivo. The authors reported > 50% transgene silencing after peptide siRNA delivery in human embryonic retinal cells in vitro, and they demonstrated that this peptide-based nanocarrier can transduce approximately 85% of the neural retina 2 h after intravitreal injection. The lack of toxicity, biodegradability, and serum stability of these nanoparticles are promising signs of a new delivery vehicle (Johnson et al. 2008).

#### Dendrimers

Dendrimers are composed of amine-containing cationic polymers such as poly-L-lysine and have been investigated for (anti-VEGF) delivery to RPE cells in vitro. They induce long-term (4–6 months) inhibition (up to 95%) of laser-induced CNV after intravitreal injection as shown in a rat model, without any observable adverse effects (Marano et al. 2005). PEI dendrimers have also been tested as a vehicle for the delivery of an shRNA expression plasmid to retinal ganglion cells via intravitreal injection. The knockdown effect was observed as early as 16 h post-injection and was sustained for 2 months (Liao and Yau 2007).

#### Gold NPs

The transfection efficiencies achieved using gold-PEI NPs in a monkey kidney cell line have been found to be several folds higher than transfecting using PEI NPs alone, although the increase in transfection efficiency was associated with decreased cell viability. More recently, Sharma et al. showed that gold-PEI NPs were effective in transfecting human corneal cells in vitro and rabbit cornea in vivo (Thomas and Klibanov 2003; Sharma et al. 2011).

## Conclusion and future perspective

In our search for new treatment options for ocular diseases, we have witnessed tremendous growth in the number of publications that present new approaches in the fields of nanomedicine and drug delivery. However, the delicate and complex anatomy of the eye is a challenge for such nanotechnology strategies. Topical application is still the most common therapeutic route for the treatment of ocular diseases, but less than 5% retinal bioavailability and often considerable ocular surface toxicity continue to be a major hurdle. A second most common problem with the application of these new approaches in the clinic is that the frequent intravitreal injections impose other risks and are not convenient for the patient. Though the design of minimally invasive, sustained drug delivery systems is challenging, this nanomedicine approach is still attractive. Novel techniques such as implants, devices, and nanoparticles possess great potential to solve the shortcomings of the currently available delivery systems (Yadav et al. 2018). As more of these therapeutic approaches come through pre-clinical research into clinical development, we anticipate the landscape of retinal therapies will change considerably, with positive effects for patients and providers alike. An important factor to be considered with implants and inserts is the obscurement of vision by the carrier. Therefore, it is recommended to use particles around 300 nm in size or smaller with careful localization. Compared to other systems, nanoparticles have been extensively studied for both anterior and posterior segment delivery (Joseph and Venkatraman 2017). However, there are only 10 nanomedicine formulations on the market for ocular disease treatment. Among them, one is indicated for glaucoma as eye drops, Timoptic-XE®, (timolol maleate ophthalmic gel forming solution, NDC 0006–3557-35 or NDC 0006–3558-35, Merck & Co. Inc.) and one for wet AMD through intravenous injection (Visudyne®, liposomal verteporfin) (Khiev et al. 2021). Moreover, according to a new commentary, nanomedicine has failed to deliver its promises, specifically in the treatment of brain and ocular diseases, mainly focusing on cancer treatment (Park 2019). In a response by Germain et al., it was clarified that the market share of ocular and brain nanomedicine and the small number of clinical trials is negligible in comparison with that of cancer and imaging agents (Germain et al. 2020). Moreover, from our point of view, this delay in clinical progress is surprising, given the fact that the first nanoparticles (PBCA) to cross the blood–brain barrier (identical to the BRB) after intravenous injection did not lead to clinical trials yet, despite the many studies which were published since 1995 (Kreuter et al. 1995). It has only achieved clinical trials for the treatment of hepatic carcinoma as the particles accumulate mainly in the liver (Wang et al. 2015). The same is true for Patisiran, the first approved siRNA therapy for the treatment of transthyretin-mediated amyloidosis after intravenous injection. The siRNA of Patisiran is loaded in lipid nanoparticles to target transthyretin-TTR, which directs it to the liver which is the primary site of TTR production (Morrison 2018). Thus, despite promising results pre-clinically, there is still a nano-drug design problem that follows the down-top approach and not vice-versa as required (Cheng et al. 2015).

Many groups have now adopted an alternative strategy by having employed smarter carriers by encapsulating the viral vectors inside a nanoparticle (Rajagopal et al. 2018; Kasala et al. 2016; Yu et al. 2021). Still, more than 100 nanomedicine start-up companies were created with products tested in > 30 clinical trials in the USA alone. Moreover, nanomedicine accounts for approximately 5% of research publications in the field of nanotechnology worldwide.

In sum, while the ocular nanomedicine field is still in a rather early stage of development, the fact that different polymeric drug delivery implants for ocular diseases have already been approved by the FDA indicates that the clinical translation of nanomedicine is still a promising road to the future of ophthalmology (Joseph and Venkatraman 2017; Park 2019). On the other hand, developing clinically effective treatments for retinal neurodegeneration will also require the delivery of specific neuroprotective agents. Glaucoma patients are expected to benefit from neuroprotective agents when diagnosed at an early stage. However, for late-stage glaucoma with severe RGCs loss, neuroprotection may not suffice. Here, other approaches like cell therapy, RGCs transplantation, and electrical/light/magnetic field stimulation require more studies as they can be used as adjuvant therapy with the available strategies (Gokoffski et al. 2020; Pardue and Allen 2018; Fudalej et al. 2021; Gall et al. 2016; Sabel et al. 2020a, b).

## Data Availability

No data were generated in-house, and no paper mill was used to help prepare the manuscript. All references are publicly available (e.g., PubMed).

## Abbreviations

AAV: Adeno-associated virus
ADRB2: B2-adrenergic receptor
AEs: Adverse events
AMD: Age-related macular degeneration
AON: Antisense oligonucleotide
ATP: Adenosine triphosphate
BAB: Blood-aqueous barrier
BBB: Blood-brain barrier
BCVA: Best corrected visual acuity
BDNF: Brain-derived neurotrophic factor
BRB: Blood-retinal barrier
CFH: Complement factor H
CFSE: Carboxyfluorescein-succinimidyl-ester
CLS-TA: Corticosteroid triamcinolone acetonide
CMV: Cytomegalovirus
CNTF: Ciliary neurotrophic factor
CNV: Choroidal neovascularization
CPP: Cell-penetrating peptide
DC-Chol: 3β-[N-(N’,N’-dimethylaminoethane) carbamoyl] cholesterol
DDAB: Dimethyl dioctadecyl ammonium bromide
DDS: Drug delivery systems
DME: Diabetic macular edema
DOPE: Dioleoylphosphatidylethanolamine
DOTAP: 1,2-Dioleoyl-3-trimethylammonium propane
DR: Diabetic retinopathy
ECM: Extracellular matrix
ECT: Encapsulated cell technology
EGFR: Epidermal growth factor receptor
FDA: Food and drug administration
FITC: Fluorescein isothiocyanate
HA: Hyaluronic acid
ICON: In-vivo confocal neuroimaging
ILM: Internal limiting membrane
IOP: Intraocular pressure
IVT: Intravitreal
K5: Kringle 5
MEMS: Microelectromechanical system
MNPs: Magnetic nanoparticles
mRNA: Messenger ribonucleic acid
NGF: Nerve growth factor
NPs: Nanoparticles
OCT: Optical coherence tomography
PAMAM: Polyamidoamine
PB: Peribulbar
PBCA: Poly(N-butyl cyanoacrylate)
PCL: Polycaprolactone
PDGF: Platelets-derived growth factor
PDT: Photodynamic therapy
PEG: Polyethylene glycol
PEI: Poly(ethylenimine)
PGA: Polyglycolide or poly(glycolic acid)
PLA: Poly(lactic acid) or polylactide
PLGA: Poly(lactide-co-glycolide) or poly(lactic-co-glycolic acid)
PNIPAAm: Poly(N-isopropylacrylamide)
PPI: Poly propylene imine
PVP: Polyvinyl-pyrrolidone
RB: Retrobulbar
RGCs: Retinal ganglion cells
RNA: Ribonucleic acid
ROS: Reactive oxygen species
RP: Retinitis pigmentosa
RPE: Retinal pigment epithelium
SC: Suprachoroidal
siRNA: Small interfering ribonucleic acid
ssDNA: Single-stranded deoxyribonucleic acid
ssRNA: Single-stranded ribonucleic acid
ST: Sub-tenon
TA: Triamcinolone acetonide
TKI: Tyrosine kinase inhibitors
TMAG: N-(alpha-trimethylammonioacetyl)-didodecyl-D-glutamate
TTR: Transthyretin
VEGF: Vascular endothelial growth factor
